# Development of the Physical Distancing Scale for COVID-19 Avoidance (PDS-C19©) in immunocompromised and non-immunocompromised individuals and its validation in the EAGLE Study

**DOI:** 10.1186/s41687-026-01150-w

**Published:** 2026-07-31

**Authors:** James C. Marcus, Tiago Maia, Paul Williams, Renata T. C. Yokota, Sofie Arnetorp, Timothy A. Herring, Sudhir Venkatesan, Philip A. Powell, Johan L. Hans Severens, James W. Varni, Sylvia Taylor, Marieke Krol, John E. Ware

**Affiliations:** 1 Patient Centered Solutions, IQVIA, Washington, DC USA; 2 Patient Centered Solutions, IQVIA, Porto Salvo, Portugal; 3 Patient Centered Solutions, IQVIA, Courbevoie, France; 4 Global Evidence, BioPharmaceuticals Medical, AstraZeneca, Gothenburg, Sweden; 5 P95 Epidemiology & Pharmacovigilance, Leuven, Belgium; 6 Health Economics & Payer Evidence, BioPharmaceuticals Medical, AstraZeneca, Gothenburg, Sweden; 7 Medical Evidence, Epidemiology, Vaccine & Immune Therapies, BioPharmaceuticals Medical, AstraZeneca, Wilmington, DE USA; 8 BPM Evidence Statistics, BioPharmaceuticals Medical, AstraZeneca, Cambridge, UK; 9 SCHARR, University of Sheffield, Sheffield, UK; 10 Philip A. Powell Consulting, Sheffield, UK; 11 Severens HTA Consultancy, Venray, Netherlands; 12 Texas A&M University, College Station, TX USA; 13 Medical Evidence, Vaccine & Immune Therapies, BioPharmaceuticals Medical, AstraZeneca, Cambridge, UK; 14 Patient Centered Solutions, IQVIA, Amsterdam, Netherlands; 15 John Ware Research Group, Watertown, MA USA

**Keywords:** Behavioral assessment scale, Clinical outcome assessment, COVID-19, EAGLE Study, PDS-C19, Physical distancing

## Abstract

**Background:**

Many immunocompromised individuals continue to practice physical distancing behaviors to avoid severe COVID-19 outcomes. To measure the intensity of these physical distancing behaviors, we aimed to develop and validate a standardized metric across age groups: the Physical Distancing Scale for COVID-19 Avoidance (PDS-C19©).

**Methodology:**

PDS-C19 development involved content scoping, initial draft PDS-C19 design phase, and exploratory testing (via asynchronous online forums). Psychometric properties (structural validity, reliability, and construct validity) of the PDS-C19 were evaluated using a random sample of immunocompromised and non-immunocompromised participants (*n* = 1059) of the EAGLE Study, including adults and children aged 0.5–17 years from the US and UK. Adult caregivers of children aged ≤ 12 years completed a proxy version of the PDS-C19 on their behalf, in addition to providing self-reported responses.

**Results:**

Findings from the two asynchronous forums (*n* = 23 and *n* = 22) indicated that the items identified in the scoping phase were relevant and well-understood. As part of the structural validity analysis, item-response distributions, inter-item correlations, and exploratory factor analyses supported a nine-item unidimensional intensity scale. Confirmatory factor analyses reduced the PDS-C19 to seven items (two items were dropped due to high residual correlations or differential item functioning by age). Internal consistency was high (ω = 0.97); convergent validity was good, with high correlations (*r*_max_=0.74) with clinical outcome assessments of the ability to participate in activities and with worry. The PDS-C19 had good known-groups validity, with scores distinguishable by types of activities, level of worry, and number of close contacts.

**Conclusion:**

The PDS-C19 had acceptable structural and construct validity and high internal consistency, demonstrating its value as a measure of physical distancing to avoid COVID-19 in immunocompromised and non-immunocompromised individuals.

**Supplementary Information:**

The online version contains supplementary material available at https://doi.org/10.1186/s41687-026-01150-w.

## Abstract (plain language summary)

Even though COVID-19 restrictions have been lifted, some people with weak immune systems still stay away from people and places to avoid getting COVID-19. This is called physical distancing. Physical distancing can make people’s lives less enjoyable and less productive. The aim of this study was to develop a survey to find out how much people are distancing. We first tested the survey on 24 people who were very vulnerable to COVID-19, including people with weak immune systems. We then gave the survey to 1059 people of all ages, both with and without weak immune systems. Caregivers completed the survey on behalf of young children. We used mathematical methods to test how well the survey worked. Based on these tests, we made improvements to the survey. The improved final survey worked well to show how much people of all ages, and with and without weak immune systems, physically distanced to avoid COVID-19.

**Supplementary Information:**

The online version contains supplementary material available at https://doi.org/10.1186/s41687-026-01150-w.

## Background

In March 2020, during the first wave of COVID-19, physical distancing was introduced by health authorities in the United Kingdom (UK) and United States (US) to help reduce the incidence of COVID-19 [1–4]. Physical distancing strategies included moving indoor activities outdoors, avoiding or reducing time spent in crowded places, asking visitors at home to maintain distance and, generally, increasing space and distance between oneself and others [3, 5].

Multiple studies conducted during mandatory COVID-19–related societal restrictions (referred to as “lockdown”) showed that physical distancing was associated with reduced health-related quality of life (HRQoL) [6–11]. Mostly through the success of global COVID-19 vaccination programs [12, 13], these lockdown restrictions were lifted by 2022 for the UK and the US [14, 15]. However, immunocompromised individuals, who comprise an estimated 3.9% of the UK adult and adolescent population [16], 2.7% to 6.6% of the US adult population [17, 18], and 2.6% of US children [18], have suboptimal vaccine responses [19–21]. Furthermore, despite representing less than 10% of the population, between 12.2% and 22% of people hospitalized for COVID-19 are immunocompromised, demonstrating that they are disproportionately impacted by COVID-19 [16, 22, 23]. Hence, as of May 2024, UK and US guidance for immunocompromised individuals continues to encourage physical distancing to avoid COVID-19 [24, 25]. However, research conducted post-lockdown indicates that immunocompromised individuals have higher levels of worry due to COVID-19 and poorer mental health outcomes than the general population, which might be influenced by COVID-19 avoidance behaviors, including physical distancing [26]. Despite this, there is a gap in the understanding of behaviors of immunocompromised individuals after the removal of lockdown restrictions and how the intensity of COVID-19 physical distancing in immunocompromised individuals relates to HRQoL. In addition to immunocompromised individuals, there are other populations who may be physically distancing (e.g., elderly, other vulnerable populations, individuals fearful of COVID-19) [27, 28].

Clinical outcome assessments (COAs), e.g., patient-reported outcomes, measure a patient’s health status or HRQoL and can help determine whether an intervention provides clinical and/or HRQoL benefit [29–40]. While physical distancing scales reported in the literature have focused on adherence to public health distancing guidelines or proxy measures of physical distancing [9, 41–45], currently no physical distancing scales have been adequately validated for use in both immunocompromised and non-immunocompromised populations, or among adolescents, children, toddlers and infants, hereafter referred to as “non-adults” (i.e., those aged < 18 years). Therefore, we developed and validated a new self- and proxy-reported behavioral instrument: the Physical Distancing Scale for COVID-19 Avoidance (PDS-C19^©^) to measure the intensity of physical distancing behaviors. The scale was initially designed to be used and validated in the EAGLE Study, a large, non-interventional, observational, cross-sectional study investigating the association between physical distancing behaviors to avoid COVID-19 and HRQoL outcome measures among immunocompromised and non-immunocompromised participants in the US and UK across all ages [46].

## Methods

### PDS-C19 content development stage (initial draft to final draft)

The PDS-C19 development comprised three phases (content scoping, initial draft PDS-C19 design phase, and exploratory testing [leading to the final draft PDS-C19]) following US Food and Drug Administration (FDA) guidance on COA development and assessment [47–51]. The PDS-C19 development and validation processes are summarized in Online Resource 1, Fig. S1.

#### Content scoping phase methodology

The PDS-C19 was designed to measure physical distancing intensity, defined as the frequency with which an individual avoids situations involving physical proximity to others to reduce the risk of COVID-19 infection. The construct focuses on distancing behavior itself, rather than related but distinct constructs such as protective behaviors, use of remote alternatives, or impairment in social roles. In this context, the construct may be defined by the following extremes: very low physical distancing intensity (lowest tendency to maintain physical space with others and thus highest risk of person-to-person transmission) and very high physical distancing intensity (highest tendency to maintain distance from others and thus lowest risk of person-to-person transmission). Physical distancing behaviors identified in the scoping phase formed the key concepts required for the PDS-C19. Protective barriers (e.g., masking) or medical interventions (e.g., vaccination) were considered conceptually distinct from the physical distancing construct because these behaviors could be undertaken without physically distancing. However, these behaviors were captured via a simple survey item in the broader EAGLE survey. Reliance on statistics to make decisions, meeting others through video apps, and using home delivery services were not considered physical distancing behaviors and were therefore not incorporated into the PDS-C19 items.

Key concepts were identified from a narrative review, conducted in April 2022 (which also aimed to identify any existing validated COVID-19–related questionnaires measuring physical distancing), a review of selected health authorities’ public health guidance aimed at high risk individuals and COVID-19 avoidance (covering the US, UK, France, Sweden, and Spain), and four online qualitative focus group sessions involving 12 participants at high risk of severe COVID-19 and two caregivers [52].

#### Content scoping phase results

No validated COVID-19–specific questionnaires measuring physical distancing were identified from the narrative review. Concepts that emerged from the health authorities’ guidance were: staying away from crowded places; avoiding close contact with others, and protective behaviors, e.g. washing hands and being vaccinated.

**Fig. 1:**
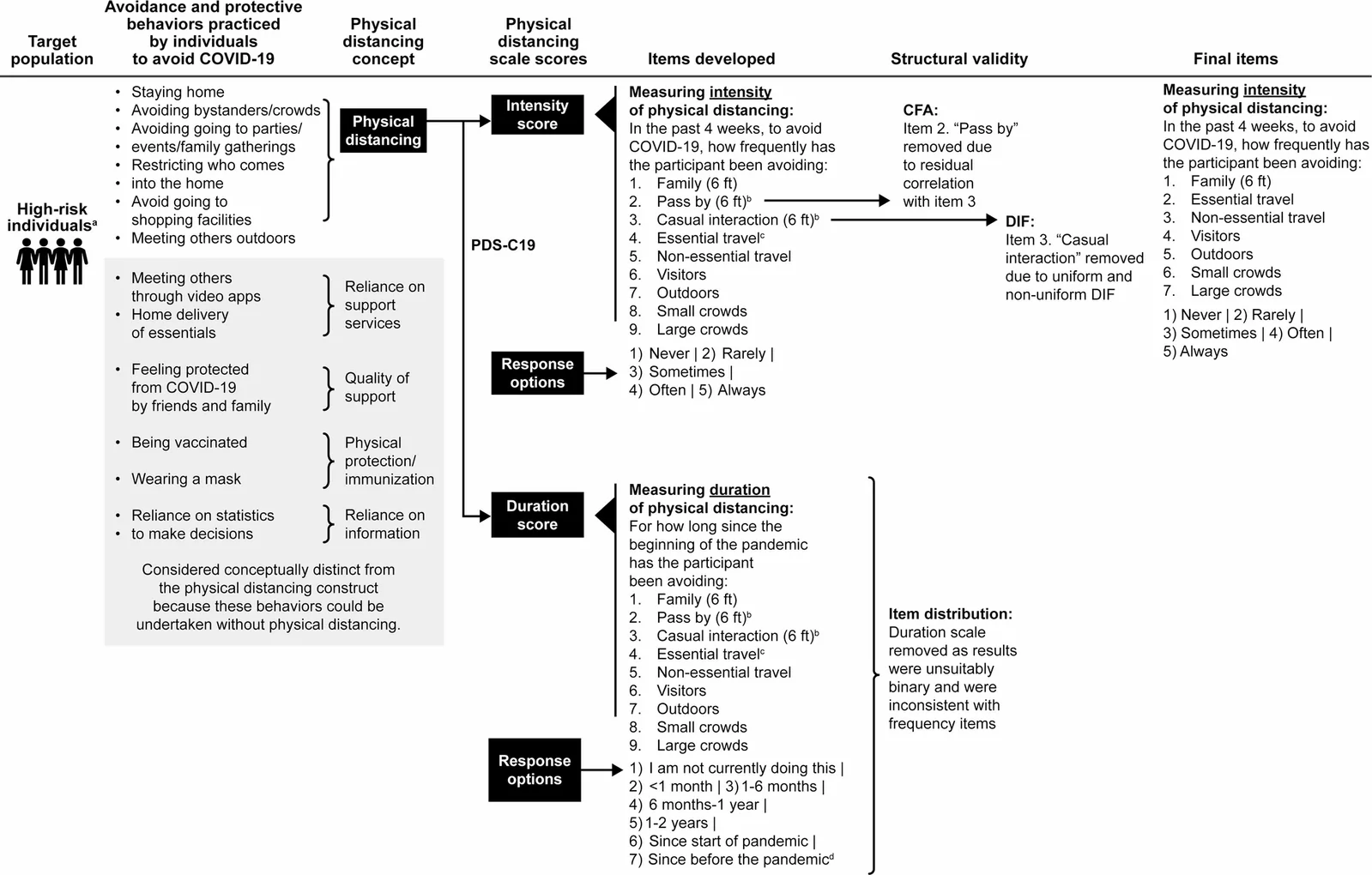
Conceptual framework of the final-draft PDS-C19. ^a^People at high risk of severe COVID-19 including those who are immunocompromised. ^b^After validation stage, item was subsequently removed from the PDS-C19 scale. ^c^After online forums, items measuring the frequency and duration of avoiding essential travel to avoid COVID-19 were added to explore how activities that may be perceived as essential, such as going to work, school, or to a healthcare appointment, may have been impacted. ^d^After online forums, an extra response option, “Before the beginning of the COVID-19 pandemic,” was added to all duration items. *COVID-19* coronavirus disease 2019; *PDS-C19* Physical Distancing Scale for COVID-19 Avoidance

Focus group participants highlighted the continued practice of physical distancing behaviors, including staying home (avoiding shopping, parties, events, and family gatherings), avoiding bystanders/crowds, restricting who comes into their home, and meeting others only outdoors, as shown in Fig. 1 [52].

#### Initial PDS-C19 design phase

The initial draft of the PDS-C19 was developed by authors experienced in scale development and psychometric validation, epidemiologists, and medical experts. Physical distancing behaviors identified during content scoping were translated into broadly applicable PDS-C19 items rather than directly converted into highly specified items (Fig. 1). For example, setting-based concepts (e.g., avoiding crowded places, shopping facilities, gatherings and outdoor contact) informed items reflecting different distancing contexts, including “Essential travel,” “Non-essential travel,” gatherings “Outdoors,” “Small crowds,” and “Large crowds.” Relationship and household-based concepts informed items on avoiding “Family” or close contacts and “Visitors.” Concepts such as masking, vaccination, use of video-based social contact, home delivery services, and reliance on statistics were excluded as they represent distinct protective, coping, or decision-making behaviors rather than physical distancing behavior itself (Fig. 1). The four-week recall period was chosen to minimize recall bias, while mitigating the influence of daily activity fluctuations on physical distancing intensity behavior traits. Furthermore, a four-week recall period is used in several other COAs; thus, use of a four-week recall period in the PDS-C19 was considered appropriate. Additional detail on the PDS-C19 initial design is in Online Resource 1, Supplementary Methods Section 1.

#### Exploratory testing phase

Two asynchronous online discussion forums were held with adult high-risk participants (including immunocompromised individuals and caregivers of individuals at high risk of severe COVID-19) from July-August 2022 (inclusion criteria in Online Resource 1, Table S1). The recruitment and outreach processes are described in Online Resource 1, Supplementary Methods Section 2.1.

The purpose of the online forums was to acquire patient and caregiver insights related to the comprehensiveness of the PDS-C19 initial draft, ascertain clarity of information, and identify possible design and interpretation problems that may cause response errors. Participant activities and the moderation process for the asynchronous forums are described in Online Resource 1, Supplementary Methods, Section 2.2.

#### Exploratory testing of the PDS-C19 initial draft

In total, there were 24 participants in the online forums; 23 attended the first forum and 21 of these participants returned in the second online forum, with one new participant (22 participants in total in the second forum). Participant characteristics are provided in Online Resource 1, Table S2.

In the first forum, most participants responded that the 16-item PDS-C19 was clear (17/23 [73.9%]), the response options were understood (16/23 [69.6%]), physical distancing behaviors at the start of the pandemic could be recalled (20/22 [90.9%]), the term “avoiding COVID-19” was clearly understood (22/22 [100.0%]), and the items applied to their experiences (19/23 [82.6%]). The reference to the specific distance of “6 feet (or 2 meters)” continued to be used as it was considered relevant for assessing physical distancing using a metric widely implemented during lockdown. Content modifications included replacing the term “meetings” with “gatherings” for the item “avoided outdoor meetings with people I do not live with.” Further details are in Online Resource 1, Supplementary Methods, Section 2.3.

Content modifications suggested during the first online forum were tested in the second online forum with 22 participants. In the second online forum, participants were asked to share their understanding of the term “physical distancing” in their own words; their opinion of the updated layout; wording of questions and response options; and their suggestions on potential additional questions. Most participants reported that they understood the term “physical distancing” (21/22 [95.5%]); the items were formatted correctly (17/22 [77.3%]); and the questions, response options, and layout were clear (21/22 [95.5%]). Further details are in Online Resource 1, Supplementary Methods, Section 2.4.

The wording and response options of the PDS-C19 were further refined, following the input from the second online forum. Two new items (i.e., frequency and duration of avoiding essential travel to avoid COVID-19) were added to complement the non-essential travel item. Additionally, an extra response option was added to all duration items, “Before the beginning of the COVID-19 pandemic,” allowing respondents to indicate pre-existing avoidance behaviors.

Thus, the final draft of the PDS-C19 had 18 items: nine measuring the frequency of physical distancing behaviors and nine measuring their duration (Fig. 1).

#### Developing the caregiver proxy scale for children

A caregiver proxy sub-version of the final draft of the PDS-C19 was created for caregivers of children aged ≤ 12 years to accommodate challenges in comprehension and potential validity of data in younger age groups and was considered appropriate for this age group, as a caregiver was expected to have a reasonable observation of the child’s daily activities and contacts. Accordingly, in this sub-version, the subject was changed to indicate the child’s behavior, i.e., from “I avoided” to “My child avoided.”

#### Final draft of the PDS-C19

The final draft of the PDS-C19 was developed to be understood by both UK and US English speakers. This draft was also translated into US Spanish using a one-step verification process, whereby the full PDS-C19 was translated into Spanish by a professional linguist, and a second linguist reviewed the translated document for accuracy, completeness, and consistency with the English language version. However, this US Spanish version was not examined with cognitive debriefing. Usability testing and screenshot review were performed to ensure the PDS-C19 was correctly displayed on all screen sizes. Full details on the final draft PDS-C19 are in Online Resource 1, Supplementary Methods, Section 3.

### PDS-C19 validation stage (final draft to final PDS-C19 and scoring)

#### Validation sample

The measurement properties (structural validity, reliability, and construct validity) of the final draft PDS-C19 were evaluated as the preliminary part of the EAGLE Study (AstraZeneca study code: D8850R00013). The full EAGLE Study methodology is reported in a protocol manuscript [46]. Briefly, participants were immunocompromised individuals, including children (aged ≥ 6 months) and their caregivers, and non-immunocompromised participants in the US and UK who reported no receipt of passive immunization against COVID-19. Participant recruitment and data collection took place from December 2022 to June 2023 using direct-to-patient channels, including panels, clinician referral, patient advocacy groups, and social media.

When counting caregiver-child dyads as two participants, 5717 eligible participants completed up to, and including, the PDS-C19 within the EAGLE survey; and 5201 fully completed the EAGLE survey. These participants comprised US- and UK-based immunocompromised and non-immunocompromised adults (≥ 18 years), adolescents (13 to 17 years), children (6 months to 12 years), and caregivers (≥ 18 years) of the non-adults. The PDS-C19 was self-reported for adults and adolescents and proxy-reported by caregivers for children ≤ 12 years; caregivers, including those of adolescents, also completed the PDS-C19 on their own behalf. Of the 5717 participants, psychometric analyses were conducted using a stratified randomly selected subset (*n* = 1059, hereafter, the “validation sample”), comprising the following ratios of key characteristics: 2:1, immunocompromised versus non-immunocompromised; 3:3:1, adult versus non-adult versus caregiver of non-adults; 1:1, male versus female; and approximately 1:1, US residents (55%) versus UK residents (45%). Details of participant flow and exclusion criteria are in Online Resource 1, Section 4 and Table S3.

No formal sample size assessment was conducted, but a sample size of approximately 1000 participants (~ 20% the total EAGLE Study) was considered reasonable for this analysis to ensure the representation of a diverse population on key characteristics throughout the range of the physical distancing intensity trait; further, it permitted splitting the sample (with stratification) into separate exploratory factor analysis (EFA) and confirmatory factor analysis (CFA) samples [53].

The initial EFA contained 542 participants, and the subsequent CFA contained 517 participants (allowing ~ 5 subjects per parameter estimated in the CFA). This validation sample far exceeded the minimum sample size (460) stipulated for CFA by Wolf et al. [53]. Differential item functioning (DIF) analyses were conducted using the CFA sub-sample. When finalizing the PDS-C19 scoring (see scoring section), scores were estimated on the centering sample (*n* = 796), a subset of the validation sample that excluded participants who responded “Never” to all final items. The scale was not validated in US Spanish, as there were only five Spanish-speaking participants.

#### Measures

The age-appropriate COAs used to assess convergent validity are described in Online Resource 1, Section 5 and Table S4 [29–40].

### Statistical Analyses

#### Statistical software

Latent variable-based analyses (EFA, CFA, DIF, latent score estimation) were performed using Mplus 8.8 (Mplus, Los Angeles, CA, USA). All other analyses were performed using SAS Enterprise Guide version 8.2 (SAS Institute, Cary, NC, USA).

#### Structural validity

##### Item-level analyses

For the PDS-C19, the item response distributions and within-scale polychoric inter-item correlations were examined.

##### Exploratory factor analysis

Following item-level analyses, EFA was conducted on the nine frequency items. The PDS-C19 was intended to be unidimensional, but given the design of the measure, a bifactor model (a general factor with uncorrelated specific factors) was also fit to account for potential nuisance factors that could emerge due to shared phrasing among groups of items (e.g., “[6 ft],” “travel,” “crowds”). The EFA model(s) were assessed via the χ^2^ test of adequate model fit (criteria: *p* > 0.05); root mean square error of approximation (RMSEA) with corresponding 90% confidence interval (CI; criteria: upper CI ≤ 0.05 to indicate close fit); standardized root mean squared residual (SRMR; criteria: ≤0.08); comparative fit index (CFI) and the Tucker-Lewis Index (TLI; criteria ≥ 0.95 for both).

##### Confirmatory factor analysis

Following EFA, the unidimensional nine-item PDS-C19 was validated via a polytomous item factor analysis. Model fit was assessed via the same criteria as EFA, with the additional steps of examining residual correlations and modification indices to identify local sources of misfit and to identify item pairs that would greatly improve the fit by allowing correlated errors.

##### Differential item functioning

To check whether people from different groups respond differently to a PDC-C19 item, even when they have the same level of the trait being measured, differential item functioning (DIF) was examined using multiple-indicator, multiple-cause models [54, 55], in which a given variable of interest was regressed on both the latent PDS-C19 score and a given item (as was the interaction between the variable and the PDS-C19 score). The variables examined were sex, country, immunocompromised status, and age group.

##### Scoring

Final calibration of the PDS-C19 was conducted using a robust maximum likelihood estimator on the centering sample. Latent trait scores were transformed to a T-score metric with a mean of 50 and standard deviation (SD) of 10, where higher scores denote greater intensity of physical distancing. For ease of scoring, while retaining the benefits of improved accuracy via latent trait scores, a T-score map was produced to provide the mean latent T-score at each raw sum score value (0–28). To provide discrete ordinal groups for characterizing physical distancing intensity, the following SD-based bands were defined on the T-score metric, similar to those defined by Cella et al., 2010 [56]: Very low, <−2 SD (< 30); Low, − 2 to − 1 SD (30 to < 40); Moderate, − 1 to 1 SD (40 to 60); High, > 1 to 2 SD (60 to 70); and Very high, > 2 SD (> 70). The average of the centering sample was labeled “moderate” allowing for application in broader samples (i.e., including individuals who report “never” distancing).

#### Reliability

##### Internal consistency reliability

Internal consistency reliability of the PDS-C19 was assessed on the centering sample as part of the CFA analysis via McDonald’s ω, a latent trait-based estimate of the proportion of variance in the unit-weighted total score attributable to all sources of common variance [57]. McDonald’s ω is bounded between 0 and 1 (higher values denote greater internal consistency).

##### Item-total correlations

Overlap-corrected item-total correlations were calculated on the centering sample using polyserial correlations between each PDS-C19 item and the latent score (estimated without the item) to provide complementary information about internal consistency, with high (*r* > 0.5) values indicating that an item contributes information to the score.

#### Construct validity

##### Convergent validity

Convergent validity was examined on the validation sample via correlations between the PDS-C19 and other COA scales collected as part of the EAGLE survey (Online Resource 1, Table S4 and Section 5). Measures that correlated to the order of 0.4–0.8 demonstrate convergent validity [58].

As maintaining physical distancing from others may constrain participation in daily activities, work or school, and social situations, PDS-C19 scores were hypothesized to correlate highest with measures of activity limitations, role functioning, social functioning, and COVID-19-related worry. Daily activity impairment and work/school presenteeism (among the employed and 13–17-year-olds) were measured by Work Productivity and Activity Impairment Questionnaire plus Classroom Impairment Questions: Specific Health Problem (WPAI-CIQ-SHP); social function and role limitations were measured by Quality of Life Disease Impact Scale-7 (QDIS-7), with items divided between HRQoL functioning and feelings; and social function was measured through the 12-item short-form survey version 2 (SF-12v2) and Pediatric Quality of Life Inventory (PedsQL). The scales that measure or include domains measuring role functioning do not refer to a willingness or a choice by the participant to avoid roles; they measure limitations in role functioning considering the participants’ circumstances. Measures of worry were captured through the Hospital Anxiety and Depression Scale (HADS) and PedsQL.

##### Known-groups validity

To assess the known-groups validity, the PDS-C19 scores were compared across levels of appropriate categorical survey items, with differences in scores between levels of each indicator assessed via analysis of variance and post hoc testing via Helmert (ordinal) contrasts [59, 60]. It was anticipated that the PDS-C19 intensity-scale scores would ordinally increase with more stringent reasons for leaving home, greater worry of serious COVID-19 illness, never having tested positive for SARS-CoV-2 infection, and more restrictive daily close contact.

### Statistical interpretation

EAGLE was a descriptive study, and no formal power calculation was performed. *P*-values < 0.05 denote nominal statistical significance unless specified otherwise (e.g., DIF analyses, post hoc known-group ordinal contrasts) and must be interpreted with caution as they do not account for multiple comparisons. Missing data are reported in tables where applicable.

## Results

### PDS-C19 validation

#### Sample characteristics

Characteristics of the 1059 participants used for the PDS-C19 validation sample are summarized in Table 1. Among those participants with reported data, most were immunocompromised (66.5% [704/1059]), and a broad range of immunocompromising conditions were represented. Of the 1059 participants, 459 comprised the adult reference group, 148 were adult caregivers (who reported data for themselves and their children), and 452 were aged < 18 years, including 152 adolescents aged 13–17 years (who reported data for themselves, albeit with support of a caregiver when needed).

**Table 1:** Characteristics of the validation sample (*n* = 1059)^a^

Characteristic	*n* (%)
**Immunocompromised status**	
Immunocompromised	704 (66.5)
Non-immunocompromised	355 (33.5)
**Immunocompromised category** ^**a**^	
Immunosuppressant treatment	171 (16.1)
Solid tumors (on active treatment)	50 (4.7)
Hematological malignancies	63 (5.9)
Primary immunodeficiency disorder	78 (7.4)
COVID-19 vaccine contraindication	82 (7.7)
Solid organ transplant	42 (4.0)
End-stage kidney disease	17 (1.6)
Stem-cell transplant or other bone marrow transplant	23 (2.2)
HIV infection (uncontrolled)	11 (1.0)
Other immunocompromising conditions	337 (31.8)
None of the above	354 (33.4)
Missing	3 (0.3)
**Sex**	
Female	546 (51.6)
**Age**	
Adult (≥ 18 years)	459 (43.3)
< 18 years	452 (42.7)
Caregivers ≥ 18 years	148 (14.0)
**Age group, years** ^**b**^	
0.5–4	73 (6.9)
5–12	227 (21.4)
13–17	152 (14.4)
18–24	41 (3.9)
25–34	107 (10.1)
35–44	155 (14.6)
45–54	124 (11.7)
55–64	94 (8.9)
≥ 65	86 (8.1)
**Marital status** ^**c**^	
Single and never married/in civil partnership	114 (10.8)
Married/in legally recognized civil partnership/long-term relationship/cohabitation	397 (37.5)
Divorced/separated/widowed	52 (4.9)
Prefer not to say	7 (0.7)
Not applicable	452 (42.7)
Missing	37 (3.5)
**Education level** ^**c**^	
High school or less	128 (12.1)
Less than a 4-year degree	139 (13.1)
4-year degree	177 (16.7)
Advanced degree	120 (11.3)
Prefer not to say	6 (0.6)
Not applicable	452 (42.7)
Missing	37 (3.5)
**Country**	
United States	584 (55.1)
United Kingdom	475 (44.9)
**Race/Ethnicity (US)** ^**a**^	
White	410 (38.7)
Black	134 (12.7)
Asian	15 (1.4)
Other^d^	54 (5.1)
Prefer not to say	3 (0.3)
Not applicable	475 (44.9)
**Race/Ethnicity (UK)** ^**a**^	
White	364 (34.4)
Asian or Asian British	37 (3.5)
Black, Black British, Caribbean, or African	41 (3.9)
Other^e^	34 (3.2)
Prefer not to say	1 (0.1)
Not applicable	584 (55.1)
**Employment status (** ***n*** ** = 607)**	
Employed full time/part time	368 (60.6)
Disabled	65 (10.7)
Retired	82 (13.5)
Unemployed	30 (4.9)
Stay at home to look after family	44 (7.2)
Prefer not to say	18 (3.0)
**Ever tested positive for COVID-19**	
Yes	317 (29.9)
**Ever hospitalized for SARS-CoV-2**	
Yes	54 (5.1)
**Number of SARS-CoV-2 vaccine doses**	
0	291 (27.5)
1–2	268 (25.3)
≥ 3	420 (39.7)
Missing	80 (7.6)
**Last positive SARS-CoV-2 test (** ***n*** ** = 317)** ^**f**^	
< 2 months ago	23 (7.3)
2–6 months ago	44 (13.9)
6–12 months ago	121 (38.2)
> 12 months ago	129 (40.7)
**Technologies used in past 4 weeks** ^**a**^	
Telephone contact with friends or family	717 (67.7)
Video call contact with friends or family	594 (56.1)
Social networking applications	469 (44.3)
Online messaging, pharmacy, or grocery service	749 (70.7)
Telephone/video healthcare	116 (11.0)
None of the above	32 (3.0)
Missing	215 (20.3)

^a^Multiple selections allowed, counts and percentages are not mutually exclusive

^b^Categories for 18 + years include caregivers

^c^Only asked to adults or caregivers

^d^Other included American Indian, Alaska Native, Native Hawaiian, other Pacific Islander, Hispanic, and other race

^e^Other included mixed or multiple ethnic groups and other ethnic group

^f^Of participants that tested positive for SARS-CoV-2

*COVID-19* coronavirus disease 2019; *HIV* human immunodeficiency virus; *N* total number of participants in the psychometric validation set; *n* number of participants who provided response; *SARS-CoV-2* severe acute respiratory syndrome coronavirus

#### Structural validity

##### Item distribution

**Fig. 2:**
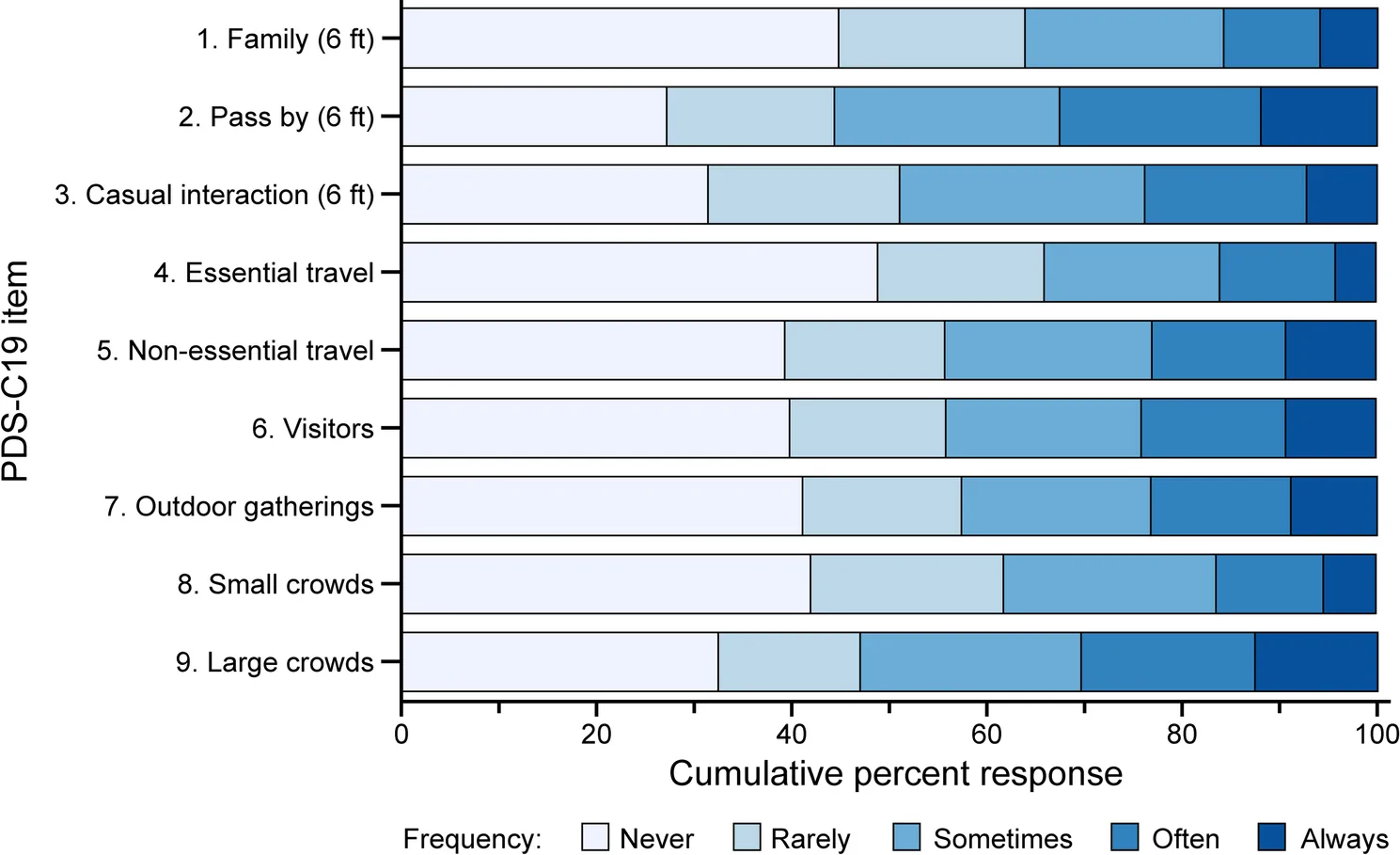
PDS-C19 item response distributions (9 frequency items), validation sample (*n* = 1059). *PDS-C19* Physical Distancing *Scale* for COVID-19 Avoidance

Item response distributions for the frequency response categories are shown in Fig. 2 for the nine-item PDS-C19 (*n* = 1059). Depending on the item, 30%–50% of the sample did not engage at all in physical distancing behaviors over the past four-week period. See Online Resource 1, Table S5 for full response distribution.

Response distributions of duration items were deemed unsuitably binary (Online Resource 1, Table S6), with most participants either “Not currently” engaging in social distancing behaviors or doing so “Since the start of the pandemic.” Additionally, duration items had inconsistent responses with the PDS-C19 frequency items, e.g., participants reported “Not currently” physically distancing but did not report that they “Never” engaged in the physical distancing behaviors assessed. Therefore, the duration scale was omitted from further psychometric analyses.

##### Inter-item correlations

Using the validation sample, polychoric correlations between the nine items ranged in the order of 0.7–0.9 (Online Resource 1, Table S7), suggesting both unidimensionality and potential redundancy. The risk of redundancy was reduced when a nine-item centering sample (*n* = 837) was used (polychoric correlations 0.5–0.8; Online Resource 1, Table S8).

##### Exploratory factor analysis (EFA)

The EFA analysis (*n* = 542) supported a single-factor model (e.g., SRMR = 0.03). A bifactor model with one general and two specific factors yielded a better fit (e.g., RMSEA improved from 0.13 to 0.09), but the specific factors were not interpretable. Therefore, only the single-factor model was carried forward to CFA (see Online Resource 1, Table S9 for complete fit statistics).

##### Confirmatory factor analysis (CFA)

In the CFA (*n* = 517), model fit was adequate for the nine items (CFI = 0.99, TLI = 0.99, RMSEA = 0.13 [95% CI, 0.12–0.15], SRMR = 0.02). However, when examining residual correlations for local misfit, the residual correlation between items 2 (“Pass by [6 ft]”) and 3 (“Casual interaction [6 ft]”) was high (0.07). Similarly, allowing the errors of items 2 and 3 to correlate (via modification index) led to a substantially greater improvement than any other pairing. Eliminating either item led to a similar improvement in model fit. Item 2 was favored for elimination as it elicited two concepts (“…6 feet of people I pass by” or “are generally nearby”), and the casual interaction evoked by item 3 was deemed easier to judge whether it had occurred. Accordingly, item 2 was dropped before the DIF analysis.

##### Differential item functioning (DIF)

As shown in Online Resource 1, Table S10, for the eight retained frequency items in the PDS-C19, item 3 (“Casual interaction [6 ft]”) was flagged for uniform and non-uniform DIF when comparing continuous age groups (≥ 65, 55–64, 45–54, 35–44, 25–34, 18–24, adolescents [13–17], older children [8–12], younger children [5–7], toddlers [2–4], and infants [< 2]), after step-up adjustment for testing eight items. These analyses also flagged item 9 ("Avoid crowded places") for uniform DIF. The items flagged for continuous age DIF were investigated via repeating their analyses using categorical age group (caregivers, adolescents, older children, younger children, toddlers, and infants), with the adult reference group. Within an item analysis, *P*-values were stepped up to account for testing six focal groups against the adult reference group. The non-uniform and uniform analyses are shown in Online Resource 1, Table S11. As a result of the observed uniform and non-uniform DIF for item 3 (“Avoid within 6 ft casual interaction”), this item was excluded from the PDS-C19 as the results implied that adults, caregivers, and non-adults (or proxies answering on their behalf) may interpret casual interaction differently. This left seven severity items, which had previously shown adequate fit during CFA. No items were flagged for DIF when assessing sex, country, or immunocompromised status.

#### Final PDS-C19 and scoring

**Fig. 3:**
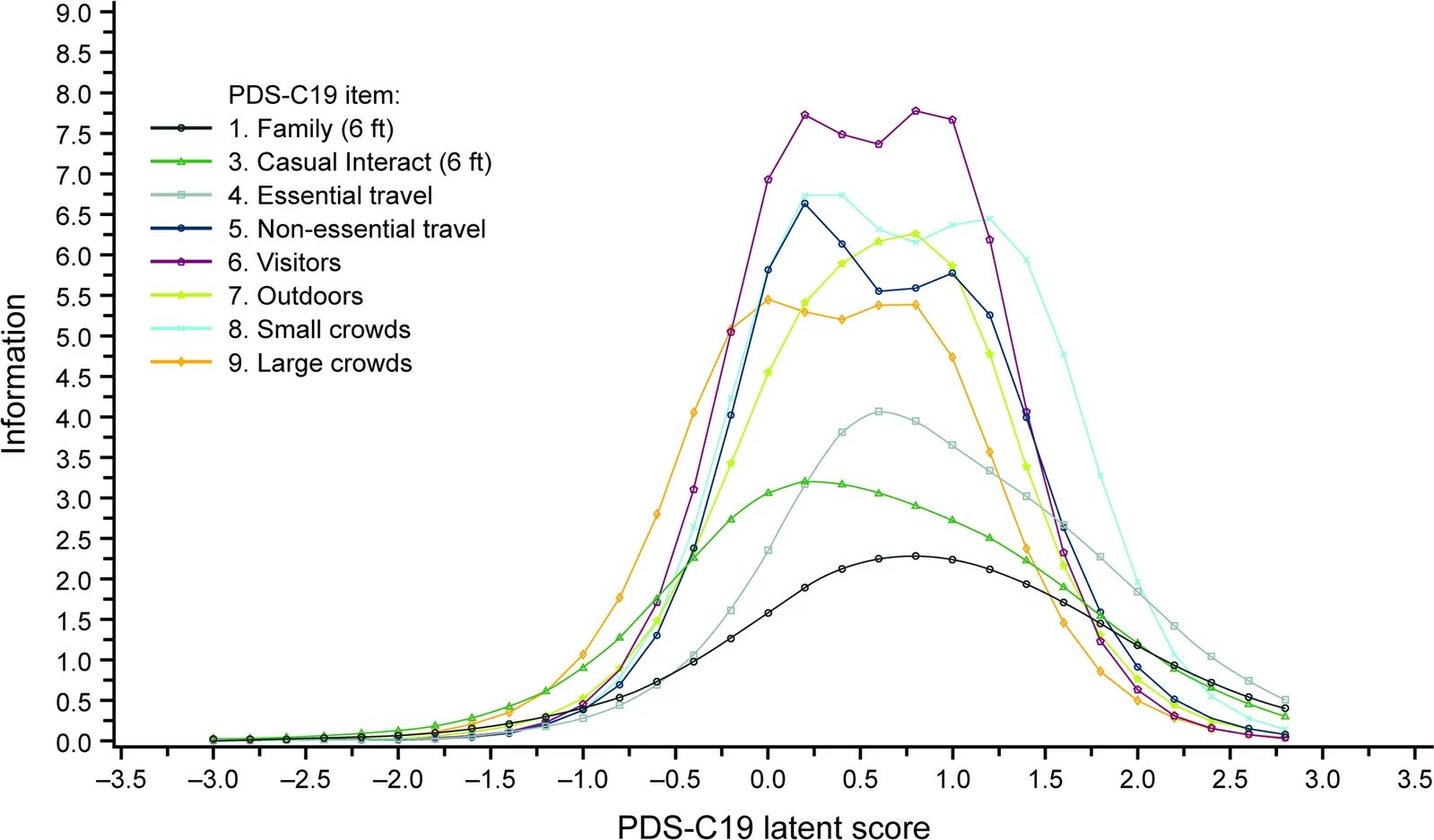
PDS-C19 item *information* curves: confirmatory factor analysis after removal of item 2. CFA subset *n* = 517. The x-axis corresponds to physical distancing intensity (i.e., the latent trait score) on a logit scale. The curves display the contribution that items make along the ability continuum. *CFA* confirmatory factor analysis; *PDS-C19* Physical Distancing Scale for COVID-19 Avoidance

When examining the item information curves (Fig. 3), item 3 (i.e., “Casual interaction [6 ft]”) provided little information about scores. Coupled with the evidence from the DIF analysis, item 3 was removed, creating the final PDS-C19. Item 1 (“Family [6 ft]”) and item 4 (“Essential travel”) also provided little information except at the upper tail (i.e., highest intensity) but were retained due to their adequate functioning overall. The seven-item final PDS-C19 yielded the following fit statistics: χ^2^ = 91.39, *p* < 0.0001; RMSEA = 0.10 (95% CI, 0.08–0.12); SRMR = 0.01; CFI = 1.00; TLI = 0.99). Model fit, as indicated by SRMR, was good, with no absolute residual correlations greater than 0.03. See Online Resource 1, Table S12 for the model fit statistics by iterative model and Online Resource 1, Table S13 for the final CFA model item loadings and thresholds. Based on all validation tests, the seven items of the final PDS-C19 were: (1) Family (6 ft): Avoiding people I care about, (2) Essential travel: Avoiding essential travel, (3) Non-essential travel: Avoiding non-essential travel, (4) Visitors: Avoiding having visitors, (5) Outdoor: Avoiding outdoor gatherings, (6) Small crowd: Avoiding places where I expect few people, (7) Large crowd: Avoiding places where I expect many people. The final PDS-C19 was available as two versions, one for people aged ≥ 13 years (self-reported version), and the caregiver proxy scale, to be given to caregivers to answer on behalf of children aged 6 months to 12 years (proxy-reported version).

##### Final scoring

The final seven-item PDS-C19 scores were calibrated using the centering sample converted to a T-score metric (mean = 50, SD = 10), where 50 represented the mean PDS-C19 score among the population of participants who engaged in at least some degree of physical distancing, with higher scores denoting greater intensity. The distribution of centering sample scores is shown in Online Resource 1, Fig. S2. A table (T-score map AstraZeneca. Data on File. 2024) was produced for mean latent T-score values at each raw sum score (PDS-C19 scoring manual. AstraZeneca. Data on File. 2024). The T-score map was used to score the PDS-C19 for outcome analyses in the EAGLE Study.

#### Reliability

##### Internal consistency reliability

The final PDS-C19 had excellent internal consistency with ω = 0.97 and overlap-corrected item-total polyserial correlations ranging from 0.61 to 0.82 (*n* = 796; see Online Resource 1, Table S14).

#### Construct validity

##### Convergent validity

As hypothesized, the PDS-C19 correlated highest with measures that assessed the ability to participate in activities and roles and measures that included attribution to COVID-19 avoidance; for example, QDIS-7, *r* = 0.74; WPAI-CIQ-SHP activity impairment, *r* = 0.62; WPAI-CIQ-SHP school presenteeism (adults and 13- to 17-year-olds enrolled in school), *r* = 0.59; WPAI-CIQ-SHP work presenteeism (employed adults), *r* = 0.49; SF-12v2 social functioning, *r* = − 0.45; and SF-12v2 role limitations due to emotional problems, *r* = − 0.45. Similarly, correlations were high with measures related to worry, e.g., PedsQL family impact module worry (caregivers, self-reported), *r* = − 0.44, and HADS anxiety score, *r* = 0.44. Pearson correlations for all measures are in Table 2.

**Table 2:** PDS-C19 convergent validity: Pearson correlations with other measures^a^, validation sample (*n* = 1059)

Scale	Measure	Sample age group completing the instrument	*n*	*r*
QDIS-7	QDIS-7 score	Adults	459	0.74
WPAI-CIQ-SHP	Activity impairment	Adults, adolescents, caregivers^b^	739	0.62
WPAI-CIQ-SHP	School presenteeism	Employed adults, adolescents, caregivers^b^	159	0.59
WPAI-CIQ-SHP	Overall school impairment	Employed adults, adolescents, caregivers^b^	159	0.58
PedsQL Infant	Psychosocial health summary score	Infants (0.5–2 years)	28	–0.57
PedsQL Infant	Total score	Infants (0.5–2 years)	28	–0.55
PedsQL Infant	Social functioning	Infants (0.5–2 years)	28	–0.55
PedsQL Infant	Emotional functioning	Infants (0.5–2 years)	28	–0.54
PedsQL Infant	Physical symptoms	Infants (0.5–2 years)	28	–0.51
PedsQL Infant	Cognitive functioning	Infants (0.5–2 years)	28	–0.51
WPAI-CIQ-SHP	Work presenteeism	Employed adults, caregivers^b^	328	0.49
PedsQL Infant	Physical health summary score	Infants (0.5–2 years)	28	–0.49
WPAI-CIQ-SHP	Overall work impairment	Employed adults, caregivers^b^	328	0.48
HADS	Depression score	Adults, adolescents, caregivers^b^	725	0.46
SF-12v2	Social functioning	Adults	459	–0.45
SF-12v2	Role limitations due to emotional problems	Adults	459	–0.45
PedsQL FIM	Worry	Caregivers^b^	148	–0.44
HADS	Anxiety score	Adults, caregivers^b^, adolescents	725	0.44
SF-6D	Short form 6-dimensions (SF-6D)	Adults	459	–0.43
PedsQL FIM	Total score	Caregivers^b^	148	–0.43
PedsQL Infant	Physical functioning	Infants (0.5–2 years)	28	–0.42
PedsQL Core	Physical functioning	Children (2–17 years)	424	–0.41
PedsQL FIM	Physical functioning	Caregivers^b^	148	–0.41
PedsQL Core	Total score	Children (2–17 years)	424	–0.40
PedsQL FIM	Parent HRQoL summary score	Caregivers^b^	148	–0.40
PedsQL FIM	Social functioning	Caregivers^b^	148	–0.40
SF-12v2	Mental component scale	Adults	459	–0.40
DMOL	How often do you feel lonely	Adults, children (8–17 years)	753	0.39
EQ-5D-5L	EQ-5D-5L health state utility	Adults, adolescents, caregivers^b^	759	–0.39
PedsQL FIM	Communication	Caregivers^b^	148	–0.39
PedsQL FIM	Family functioning summary score	Caregivers^b^	148	–0.38
SF-12v2	Role limitations due to physical problems	Adults	459	–0.38
PedsQL FIM	Daily activities	Caregivers^b^	148	–0.36
PedsQL FIM	Family relationships	Caregivers^b^	148	–0.35
PedsQL Generic Core	Psychosocial health summary score	Children (2–17 years)	424	–0.35
SF-12v2	Physical functioning	Adults	459	–0.34
SF-12v2	Mental health	Adults	459	–0.34
PedsQL FIM	Emotional functioning	Caregivers^b^	148	–0.33
WPAI-CIQ-SHP	Work absenteeism	Employed adults, caregivers^b^	331	0.33
WPAI-CIQ-SHP	School absenteeism	Employed adults, adolescents, caregivers^b^	161	0.32
PedsQL Generic Core	Emotional functioning	Children (2–17 years)	424	–0.32
SF-12v2	Bodily pain	Adults	459	–0.32
EQ-5D-Y VAS	Visual analogue scale	Children (5–12 years)	227	–0.31
PedsQL Generic Core	Social functioning	Children (2–17 years)	424	–0.31
SF-12v2	Physical component scale	Adults	459	–0.30
PedsQL FIM	Cognitive functioning	Caregivers^b^	148	–0.30
PedsQL Generic Core	School functioning	Children (2–17 years)	404	–0.30
SF-12v2	General health perceptions	Adults	459	–0.29
EQ-5D VAS	Visual analogue scale	Adults, adolescents, caregivers^b^	759	–0.29
EQ-5D-Y	EQ-5D-Y health state utility	Children (5–12 years)	227	–0.27
SF-12v2	Energy and vitality	Adults	459	–0.26

^a^Ordered by absolute *r* value. ^b^Self-reported. *DMOL* Direct Measure of Loneliness; *EQ-5D-5L* five-level version of the EQ-5D; *EQ-5D-Y* child-friendly version of the EQ-5D; *FIM* family impact module; *HADS* Hospital Anxiety and Depression Scale; *HRQoL* health-related quality of life; *PDS-C19* Physical Distancing Scale for COVID-19 Avoidance; *PedsQL* Pediatric Quality of Life Inventory; *QDIS-7* Quality-of-Life Disease Impact Scale 7; *SF-6D* Short Form 6-Dimensions; *SF-12v2* 12-Item Short Form Survey Version 2; *VAS* Visual Analogue Scale; *WPAI-CIQ-SHP* Work Productivity and Activity Impairment plus Classroom Impairment Questions: Specific Health Problem

##### Known-groups validity

Five questionnaire items with nominal/ordinal categories served as known-group variables. Table 3 provides omnibus F-tests, comparing the least squares mean of the levels of the known-group variables. Table 4 provides post hoc ordinal (Helmert) contrasts. As expected, participants who left home solely for health reasons had higher PDS-C19 scores than those who left for other reasons. For serious COVID-19 illness worries, all ordinal contrasts were statistically significant; those reporting greater worry (“Extremely worried or very”’ versus “Somewhat or less,” “Somewhat or a little” versus “Not very or less,” and “Not very” versus “Not at all”) had higher PDS-C19 scores. For daily close contact, those who had no close contact had higher PDS-C19 scores than those who had contact with at least one individual, and those who had close contact with one to five individuals had higher PDS-C19 scores than those who had close contact with six or more. However, PDS-C19 scores did not differ between those who reported ever testing positive for SARS-CoV-2 and those who had not.

**Table 3:** PDS-C19 known-groups validity: Omnibus F-test of PDS-C19 score differences^a^ validation sample (*n* = 1059)^b, c^

Groups	*n*	LS mean	SE	*P*-value
**“In the past for 4 weeks, for which of the** **following reasons did you leave your home?”**				< 0.0001
Did not leave home	14	45.12	3.17	
Health-related reasons^d^	74	55.75	1.38	
Leisure activities^e^	931	43.53	0.39	
**“Are you worried about getting seriously** **ill from COVID-19?”**				< 0.0001
Extremely or very worried	244	54.36	0.65	
Somewhat or a little worried	277	44.93	0.61	
Not very worried	209	37.72	0.70	
Not at all worried	121	35.23	0.92	
**“Are your worried about any of your friends** **and family getting seriously ill from COVID-19?”**				< 0.0001
Extremely	73	54.47	1.22	
Very	90	50.58	1.10	
Somewhat	132	42.75	0.91	
Not very	95	38.88	1.07	
Not at all	39	34.83	1.68	
**“Have you ever tested positive for an infection** **of SARS-CoV-2, the virus that causes COVID-19?”**				0.084
Never tested positive	569	45.32	0.52	
Had a positive test	317	43.81	0.70	
**“On a typical day (excluding days in** **paid employment), how many people do you** **come into close contact (under 6 feet or 2 meters)?”**				< 0.0001
0	64	48.51	1.51	
1–5	527	45.88	0.53	
6–20	246	40.52	0.77	
21–99	64	41.65	1.51	
≥ 100	27	45.82	2.32	

^a^By survey question category level

^b^All questions for adults and caregivers were self-reported. All questions for older children (8–12 years), younger children (5–7 years), infants (0.5–2 years), and toddlers (2–4 years), were caregiver-reported. For adolescents (13–17 years), some questions were self-reported such as “In the past for 4 weeks, for which of the following reasons did you leave your home?” and “Are you worried about getting seriously ill from COVID-19?” were self-reported, while other questions were caregiver reported, such as “Has your child ever tested positive for an infection of SARS-CoV-2, the virus that causes COVID-19?”. The response options for “Serious COVID-19 illness worry” differed by age group, i.e., adults: extremely, very, somewhat, not very, not at all; adolescents (13–17 years) and older children (8–12 years): very, a little, not very, not at all; younger children (5–7 years), toddlers (2–4 years), and infants (0.5–2 years), which were caregiver-reported: extremely, very, somewhat, not very, not at all. The “Are you worried about any of your friends and family getting seriously ill from COVID-19?” question was only asked to adults (18 years and older). The caregiver proxy versions substituted “Are you” with “Is your child.”

^c^Due to different questions/responses for different age groups (as described in footnote a) the categories have fewer than 1059 participants

^d^Pharmacy, hospital, health care appointment/center. These are individuals who did not select any non-health reasons

^e^Fun day out, party, organized group activity. These are individuals who may have also selected health reasons

*COVID-19* coronavirus disease 2019; *LS* least squares; *PDS-C19* Physical Distancing Scale for COVID-19 Avoidance; *SE* standard error

**Table 4:** PDS-C19 known-groups validity: Helmert contrasts of question category levels, validation sample (*n* = 1059)

Groups^a^	Mean difference	95% CI	*P*-value^b^
**Reason for leaving home**			
Did not leave home vs. any	−4.52	−10.90 to 1.87	0.166
Health related vs. leisure	12.22	9.40 to 15.03	< 0.0001
**Serious COVID-19 illness worry**			
Extremely or very vs. somewhat or less	15.06	13.53 to 16.59	< 0.0001
Somewhat or a little vs. not very or less	8.46	6.81 to 10.10	< 0.0001
Not very vs. not at all	2.49	0.22 to 4.75	0.031
**Serious COVID-19 illness worry (friends and family)**			
Extremely vs. very or less	12.71	10.02 to 15.40	< 0.0001
Very vs. somewhat or less	11.76	9.16 to 14.36	< 0.0001
Somewhat vs. not very or less	5.90	3.25 to 8.55	< 0.0001
Not very vs. not at all	4.05	0.14 to 7.96	0.043
**Ever tested positive**			
Yes vs. no	1.51	−0.20 to 3.23	0.084
**Daily close contact (people)**			
0 vs. ≥ 1	5.04	1.75 to 8.33	0.011
1–5 vs. ≥ 6	3.22	1.07 to 5.36	0.011
6–20 vs. ≥ 21	−3.22	−6.33 to − 0.11	0.083
21–99 vs. ≥ 100	−4.17	−9.61 to 1.27	0.133

^a^Please refer to Table 3 for details on variables used

^b^*P*-values within a known-groups validity analysis were adjusted for multiplicity via Holm-Sidak

*CI* confidence interval; *COVID-19* coronavirus disease 2019; *PDS-C19* Physical Distancing Scale for COVID-19 Avoidance

## Discussion

The PDS-C19 is a new self- and -proxy-reported behavioral instrument that has been designed to measure the intensity of physical distancing behaviors to avoid COVID-19 in immunocompromised and non-immunocompromised adults and children in the US and UK. Favorable cross-sectional psychometric properties have been shown in a large sample (*n* = 1059) of immunocompromised and non-immunocompromised participants covering a broad categorical age range (0.5 to ≥ 65 years). Scientific robustness was applied to PDS-C19 development and validation following US FDA COA guidance [47–50]. The excellent internal consistency of the final PDS-C19 (ω = 0.97) indicates strong coherence among items measuring a unidimensional physical distancing intensity construct. This high reliability should also be interpreted alongside the high inter-item correlations and overlapping item information curves, which suggest some conceptual and empirical overlap among items, with some items contributing information mainly at higher levels of distancing intensity. Retaining these items supported content coverage of distinct distancing contexts identified during scale development. The PDS-C19 was patient informed, strengthening its suitability for use among high-risk populations. Other physical distancing scales have been used to measure adherence to prevention strategies during pandemics [41], to compare physical distancing strategies in immunocompromised adults with non-immunocompromised adults during the COVID-19 pandemic [9], and to examine what influenced adherence to physical distancing during the COVID-19 pandemic [42–45] (Online Resource 1, Table S15).

The PDS-C19 is the first instrument designed to assess voluntary physical distancing behaviors rather than adherence to guidelines. Further, the PDS-C19 is the first scale to be designed outside the scope of mandatory COVID-19–related restrictions, psychometrically validated in immunocompromised and non-immunocompromised populations, and inclusive of all ages. As COVID-19 becomes an endemic disease, the PDS-C19 will likely be particularly useful in future research that involves immunocompromised individuals, owing to their aforementioned suboptimal responses to COVID-19 vaccines [19–21]. Furthermore, studies published after the lifting of COVID-19–related restrictions have shown that immunocompromised individuals continue to be disproportionately affected by COVID-19, compared with non-immunocompromised populations [61, 62]. While the scale has been designed and validated for use within the context of COVID-19, validation occurred outside COVID-19–related restrictions; therefore, the scale could also be adopted for use as an unvalidated measure of physical distancing behaviors to avoid other infectious diseases. Again, this could be particularly relevant for immunocompromised individuals, who have also been shown to have suboptimal responses to influenza vaccines [63]. Another consideration is that while the proxy version of the PDS-C19 is validated for use among caregivers of children, the scale could also be suitable for other types of proxies, such as caregivers of adult individuals who have difficulties with comprehension or non-English speakers, although validation has not been undertaken in these populations.

The measurement properties of the PDS-C19 have some limitations. Firstly, the EAGLE Study’s cross-sectional study design did not permit assessing the PDS‑C19’s longitudinal measurement properties: test-retest reliability, sensitivity to change over time, or evaluating a meaningful within-person change threshold. In assessing convergent validity, as hypothesized, the PDS-C19 correlated highest with measures of ability to participate in roles and activities. However, among measures of that construct, correlations with PDS-C19 were highest for those that specifically referenced avoiding COVID-19 (e.g., correlations were higher with QDIS-7 [*r* = 0.74] and WPAI activity impairment [*r* = 0.62] than they were for generic measures like SF-12v2 social functioning [*r = −* 0.45] and role limitations due to emotional problems [*r* = 0.45]). The exact influence of the reference to avoiding COVID-19 is difficult to disentangle from other aspects of the measures. For example, the correlations may be higher because a seven-item measure QDIS-7 is a better proxy of the latent trait than the one- or two-item SF-12v2 domains, and because WPAI activity impairment is a very targeted measure (“…ability to do your regular daily activities, other than work at a job or attending classes”).

Another limitation was that the PDS-C19 was only validated in US/UK English, as there were only five participants who completed the EAGLE Study survey in Spanish. Additionally, while children and their caregivers were included in the validation sample, the PDS-C19 content was not developed with input from children or from caregivers on behalf of children; the extent to which the instrument’s content comprehensively captures child and infant behaviors is not known. However, since the content was developed to include generic activities that reflect a unidimensional construct of physical distancing intensity, we may speculate that input from children would not have altered the final PDS-C19 content or structure. This scale is congruent with advice for children and caregivers of children regarding physical distancing behaviors to avoid COVID-19, and we found no empirical evidence of DIF across age groups in the final PDS-C19. Nonetheless, concept elicitation and cognitive debriefing in children and caregivers is recommended to provide additional evidence of appropriateness in non-adults. Finally, while the PDS-C19 is designed and validated for use in immunocompromised and non-immunocompromised populations, the scale may not be suitable for some sub-populations, such as those who do not have full freedom of movement (e.g., people who are hospitalized or imprisoned).

The cross-sectional nature of the EAGLE Study did not permit the assessment of test-retest reliability or the longitudinal properties of responsiveness and thresholds for within-subject clinically meaningful change for the PDS-C19. Future psychometric evaluation of the PDS-C19 could therefore look to assess these properties as well as consider the use of the PDS-C19 beyond COVID-19, i.e., for other conditions where patients may need to physical distance to reduce their risk of infection. While the seven-item measure is not perceived to be burdensome, other future analyses could be undertaken to determine whether an abbreviated version of the PDS-C19 would retain adequate reliability, validity, and content coverage while reducing respondent burden. Future studies could also explore whether the PDS-C19 would be suitable for children under 13 years of age to complete themselves. For example, in the broader EAGLE survey, the EQ-5D-Y, the PedsQL Generic Core, and the DMOL questionnaires were completed by children aged 8–12 years, and children aged 5–7 years completed the PedsQL Generic Core with support of a caregiver. Thus, it is feasible that children younger than 13 years could complete the PDS-C19 themselves, but this would need to be assessed prior to implementation.

## Conclusion

The PDS-C19 is a reliable and validated scale that has been designed to measure the intensity of physical distancing to avoid COVID-19 with a four-week recall period. This scale has been validated in an immunocompromised population and a non-immunocompromised population across a wide age range, with evidence of measurement invariance across these groups. Using the PDS-C19 in observational studies or clinical trials can lead to a better understanding of how populations physically distance to avoid COVID-19 and help determine the relationship between physical distancing and health outcomes. Further, the scale can be used to assess the broader extent of the COVID-19 burden and aid any future decision-making during the appraisal of new interventions in high-risk populations, such as the immunocompromised community.

## Supplementary Information

Below is the link to the electronic supplementary material.


Supplementary Material 1


## Data Availability

Data underlying the findings described in this manuscript may be obtained in accordance with AstraZeneca’s data sharing policy described at https://astrazenecagrouptrials.pharmacm.com/ST/Submission/Disclosure. Data for studies directly listed on Vivli can be requested through Vivli at www.vivli.org. Data for studies not listed on Vivli could be requested through Vivli at https://vivli.org/members/enquiries-about-studies-not-listed-on-the-vivli-platform/. AstraZeneca Vivli member page is also available outlining further details: https://vivli.org/ourmember/astrazeneca/. Any researchers and others interested in obtaining permission to use the PDS-C19 should contact https://www.astrazeneca.com/patient-reported-outcomes.html.

## Abbreviations

CFA: Confirmatory factor analysis
CFI: Comparative fit index
CI: Confidence interval
COA: Clinical outcome assessment
COVID-19: Coronavirus disease 2019
DIF: Differential item functioning
DMOL: Direct Measure of Loneliness
EFA: Exploratory factor analysis
EQ-5D-5L: Five-level version of the EQ-5D
EQ-5D-Y: Child-friendly version of the EQ-5D
FDA: Food and Drug Administration
FIM: Family impact module
HADS: Hospital Anxiety and Depression Scale
HIV: Human immunodeficiency virus
HRQoL: Health-related quality of life
LS: Least squares
n: Number of participants who provided response
N: Total number of participants in the psychometric validation set
PDS-C19: Physical Distancing Scale for COVID-19 Avoidance
PedsQL: Pediatric Quality of Life Inventory
QDIS: Quality-of-Life Disease Impact Scale
RMSEA: root mean square error of approximation
SARS-CoV-2: Severe acute respiratory syndrome coronavirus
SE: Standard error
SF-12v2: 12-Item Short Form Survey Version 2
SF-6D: Short Form 6-Dimensions
SRMR: Standardized root mean squared residual
TLI: Tucker-Lewis Index
UK: United Kingdom
US: United States
VAS: Visual Analogue Scale
WPAI-CIQ-SHP: Work Productivity and Activity Impairment Questionnaire plus Classroom Impairment Questions: Specific Health Problem
