# Supplementary material for: Development of the Physical Distancing Scale for COVID-19 Avoidance (PDS-C19©) in immunocompromised and non-immunocompromised individuals and its validation in the EAGLE Study
Source: J Patient Rep Outcomes. 2026 Jul 31;10:157. doi: 10.1186/s41687-026-01150-w (PMC13525139; doi:10.1186/s41687-026-01150-w)
Supplement: Supplementary file 1 [file 41687_2026_1150_MOESM1_ESM.docx]

**Online resource 1**

**Development of the Physical Distancing Scale for COVID-19 Avoidance (PDS-C19^©^) in immunocompromised and non-immunocompromised individuals and its validation in the EAGLE Study**

Table of Contents

[Supplementary Methods	3](#_Toc173313845)

[1. Initial PDS-C19 design phase	3](#_Toc173313846)

[2. Exploratory testing phase	3](#_Toc173313847)

[2.1 Asynchronous forum recruitment	3](#_Toc173313848)

[2.2 Asynchronous forum additional methods	4](#_Toc173313849)

[2.3 Findings related to suggested additional items and PDS-C19 structure: forum 1	4](#_Toc173313850)

[2.4 Findings related to suggested additional items and PDS-C19 structure: forum 2	4](#_Toc173313851)

[3. Final draft of the PDS-C19: implementation in the EAGLE Study	4](#_Toc173313852)

[4. EAGLE Study participant flow	5](#_Toc173313853)

[5. Description of clinical outcome assessments	6](#_Toc173313854)

[Table S1. Asynchronous forum inclusion and exclusion criteria	9](#_Toc173313855)

[Table S2. Characteristics of participants in the asynchronous forums (*n* = 24)	10](#_Toc173313856)

[Table S3. EAGLE Study inclusion and exclusion criteria	11](#_Toc173313857)

[Table S4. Clinical outcome assessments used in the EAGLE Study by age	12](#_Toc173313858)

[Table S5. Frequency item response distribution for the final draft of the PDS-C19 (frequency of avoidant behaviors), validation sample (*n* = 1059)	13](#_Toc173313859)

[Table S6. Duration item response distribution for the final draft of the PDS-C19 (onset of current avoidant behaviors), validation sample (*n* = 1059)	14](#_Toc173313860)

[Table S7. Polychoric correlations between the nine final-draft PDS-C19 frequency items, validation sample (*n* = 1059)	15](#_Toc173313861)

[Table S8. Polychoric correlations between the nine final-draft PDS-C19 frequency items (excluding those who answered “Never” to all items, *n* = 837)^a^	16](#_Toc173313862)

[Table S9. EFA – fit statistics of the nine-item final draft of the PDS-C19, EFA sample (*n* = 542)	17](#_Toc173313863)

[Table S10. MIMIC model for differential item functioning analysis for final-draft PDS-C19 intensity item 3 (Casual interaction [6 ft]) by age group (Reference: Adult), CFA sample (*n* = 517)	18](#_Toc173313864)

[Table S11. PDS-C19 CFA of the final draft of the PDS-C19 – fit statistics for selected items, CFA sample (*n* = 517)	19](#_Toc173313865)

[Table S12. CFA: WLSMV standardized item loadings and thresholds of the final draft of the PDS-C19, confirmatory factor analysis sample (*n* = 517)	20](#_Toc173313866)

[Table S13. PDS-C19 internal consistency reliability: Overlap-corrected item-total polyserial correlations (centering sample, i.e., excluding those who answered “Never” to all items, *n* = 796)	21](#_Toc173313867)

[Table S14. Summary of physical distancing scales used in other studies	22](#_Toc173313868)

[Fig. S1. Overview of development and validation stages of the PDS-C19	23](#_Toc173313869)

[Fig. S2. T-score distribution of seven final PDS-C19 frequency items (centering sample, i.e., excluding those who answered “Never” to all items, *n* = 796)	24](#_Toc173313870)

[References	25](#_Toc173313871)

# Supplementary Methods

## 1. Initial PDS-C19 design phase

Specific physical distancing behaviors that were identified in the scoping phase were generalized into items that could be broadly applied. For example, “avoid going to theatre” became “avoid going to places where there are expected to be many people.” This process resulted in eight items intended to distinguish between very low physical distancing intensity (lowest withdrawal from normal life and highest risk of person-to-person transmission) and very high physical distancing intensity (highest withdrawal from normal life and lowest risk of person-to-person transmission). A five-point Likert scale (“Never,” “Rarely,” “Sometimes,” “Often,” and “Always”) was incorporated to allow respondents to indicate how often they had practiced each behavior over the past four weeks (frequency). The four-week recall period was chosen to minimize recall bias, while considering the mitigating influence of daily activity fluctuations on physical distancing intensity behavior traits, and to reduce practical issues related to the administration of diary assessments in research. The Likert scale was chosen to more appropriately reflect individuals’ behavioral traits over the recall period than that of a numeric spectrum of responses (e.g., 0, 1, 2, ≥3 times) in the four-week period, the latter of which may be more prone to momentary fluctuations. In addition to being able to indicate the frequency of a physical distancing behavior, each item had a second scale to indicate the duration of the behavior at its current level: less than 1 month, between 1 and 6 months, between 6 months and 1 year, between 1 and 2 years, and beginning of the pandemic. Therefore, the initial draft PDS-C19 contained 16 items (eight frequency items to measure physical distancing intensity in the past four weeks; each item had a second scale to indicate the duration of the behavior at its current level) that were formatted into a grid structure with two layout options: in layout 1, the frequency and duration of physical distancing behaviors were presented side by side within the same table, and in layout 2, the frequency and duration of physical distancing behaviors were presented separately.

## 2. Exploratory testing phase

### 2.1 Asynchronous forum recruitment

Asynchronous forums were conducted via AstraZeneca’s Virtual Engagement Community. Participants who were considered eligible for participation were invited to participate by email, asked to provide consent for their participation, and paid an honorarium for their time participating in the online forums. Both online forums were moderated by two researchers with experience in survey development and qualitative interviews across a wide range of therapeutic areas.

### 2.2 Asynchronous forum additional methods

During the asynchronous forum, participants were first asked to read through the EAGLE survey (including PDS-C19 and other COAs) and then asked a set of structured and unstructured questions on the PDS-C19 content. Forum moderators, TM and PW, used cognitive probes to inquire into participants’ responses and comments. Each online forum was open for one week. Data generated during these online forums were summarized in tabular and narrative summary format, with supportive quotes. Categorical data generated from the responses obtained were descriptively analyzed as frequencies and percentages using Microsoft Excel^®^.

### 2.3 Findings related to suggested additional items and PDS-C19 structure: forum 1

Approximately half (12/23 [52.2%]) of the asynchronous forum participants reported that new items related to work and emotional impacts could be introduced. However, these reflected HRQoL outcomes experienced by the participants and were not directly related to the physical distancing behavior concept. Therefore, such new items were not further considered for the refinement of the PDS-C19. Both layout 1 and layout 2 of the initial PDS-C19 were presented in the first online forum, but participants agreed that layout 1 was confusing and layout 2 was clearer. Participants noted that layout 2 could be further streamlined by unmerging the response option columns so that each item had its own set of response options clearly presented and improved by refining the wording.

### 2.4 Findings related to suggested additional items and PDS-C19 structure: forum 2

In the second forum, only the refined PDS-C19 layout 2 was shown to participants. A few participants (3/22 [13.6%]) suggested that new items could be introduced related to coping strategies and mechanisms for maintaining contact with others and accessing services. These suggestions were not directly related to the physical distancing behavior concept and therefore were not considered for refinement of the PDS-C19.

## 3. Final draft of the PDS-C19: implementation in the EAGLE Study

The final draft of the PDS-C19 was developed so that a single version would be understood by both UK and US speakers of English. This draft was translated into US Spanish for use in the US portion of the EAGLE Study. The instructional language was designed to be simple, short, and appropriate for electronic administration.

The original US/UK English version and translated US Spanish sub-version were migrated onto a web-based electronic platform (compatible with mobile phones, tablets, and computers) for use within the EAGLE Study. The PDS-C19 underwent a screenshot review and usability testing to ensure the electronic version displayed and functioned correctly and that the instructions, questions, and response options were rendered correctly regardless of screen size.

## 4. EAGLE Study participant flow

A total of 22,392 participants were screened for eligibility into the EAGLE Study. The main reasons for non-eligibility were: the full quota for non-immunocompromised participants was met (9515 participants, 42.5%); the informed consent form was not completed (2203 participants, 9.8%); study participation was declined (1970 participants, 8.8%); and other reasons, including not meeting specific inclusion criteria. There were 5717 eligible participants (when counting caregiver-child dyads as two participants), who had completed up to, and including, the PDS-C19 within the EAGLE survey; 5201 of these participants fully completed the survey. Of the 5717 who had completed up to and including the PDS-C19, 1059 were included in the validation sample.

## 5. Description of clinical outcome assessments

**12-Item Short Form Survey Version 2 (SF-12v2):** The SF-12v2^®^ is a self-reported outcome instrument assessing the well-being of an individual over the past four weeks [1]. There are eight domains in the SF-12v2. The eight domain scores can be further aggregated (via factor score coefficients) into two composites: the physical and mental component scores, with norm-based scores with a mean of 50 and standard deviation of 10 derived from the US general population.

**Quality-of-Life Disease Impact Scale 7 (QDIS-7):** The QDIS-7^®^ is a generic fixed-length disease-specific HRQoL instrument that measures a unidimensional disease impact score in relation to norms determined for the chronically ill general US population [2]. It was originally derived from the 49-item QDIS full item bank, with the specific items chosen such that the following seven of the 10 content areas would be presented: 1 Quality of life; 2 Health outlook; 3 Physical functioning; 4 Role functioning; 5 Social activity; 6 Fatigue; and 7 Emotional.

**Direct Measure of Loneliness (DMOL):** The Direct Measure of Loneliness is a single-item instrument, which asks individuals directly about their experience of loneliness: “How often do you feel lonely?” [3]. Response categories are: “Often or always,” “Some of the time,” “Occasionally,” “Hardly ever,” or “Never” [3]. There is no specific recall period, with the individual providing their experience in the moment of survey completion.

**Pediatric Quality of Life Inventory™ (PedsQL™)** **Generic Core Scales:** The PedsQL™ Generic Core Scales (Mapi Research Trust, Lyon, France) is a brief measure of HRQoL in children and adolescents, with a four-week recall period [4]. Different variants exist, depending on the age of the individual and whether or not the measure is being completed as a self-report or by a parent as a proxy measure. The 23 items in the PedsQL™ comprise four Generic Core Scales: Physical Functioning (eight items); Emotional Functioning (five items); Social Functioning (five items); and School Functioning (five items).

**PedsQL™ Infant Scales:** The PedsQL™ Infant Scales is a specific module of the PedsQL™ and is used for measuring parents’ perceptions of their infant’s (aged 1 to 24 months) generic HRQoL [5]. The infants aged 1 to 12 months version comprises 36 items, and the infants aged 13 to 24 months version comprises 45 items (i.e., the 36 items in the aged 1 to 12 months version plus nine additional items) [5].

**PedsQL™ Family Impact Module (FIM):** The PedsQL™ FIM^®^ is a 36-item instrument for measuring the impact of pediatric chronic health conditions on parents and the family [6]. The PedsQL™ FIM comprises the following six domains with number of items measuring parent self-reported functioning: 1 Physical functioning (six items); 2 Emotional functioning (five items); 3 Social functioning (four items); 4 Cognitive functioning (four items); 5 Communication (three items); and 6 Worry (five items). There are the following two scales included which measure parent-reported family functioning: 1 Daily activities (three items) and 2 Family relationships (five items).

**EQ-5D-5L:** The EQ-5D-5L^®^ is the five-level version of the EQ-5D, a standard measure of health utility on the day of administration, providing a simple, generic measure of health for clinical and economical appraisal [7]. The tool comprises two components – a descriptive system and a visual analogue scale (VAS). When the descriptive system is linked to a value set, utility scores can be derived via responses to the following five items: 1 Mobility; 2 Self-care; 3 Usual activities; 4 Pain/discomfort; and 5 Anxiety/depression.

**EQ-5D-Y:** The EQ-5D-Y^®^ is the child-friendly version of the EQ-5D, developed by the EuroQol Group [8]. The EQ-5D-Y is a more comprehensible instrument suitable for children and adolescents, available in a self-report version recommended for ages 8 to 11 years and proxy versions (proxy 1 and proxy 2) recommended for ages 4 to 7 years [8]. For children and adolescents aged 12 years or older, both the EQ-5D-Y and adult EQ-5D-5L are considered appropriate.

**Short Form 6-Dimensions (SF-6D):** The SF-6D is a reference-based single index measure of health that can be calculated from items of the SF-12v2 instrument [9]. Calculation of the SF-6D from the SF-12v2 provides a method to calculate health state utilities in addition to the EQ-5D instrument and from a broader set of domains.

**Work Productivity and Activity Impairment Questionnaire plus Classroom Impairment Questions: Specific Health Problem (WPAI+CIQ:SHP):** The WPAI+CIQ:SHP is a generic work productivity and activity impairment instrument that can be adapted to various health conditions or concerns [10]. The WPAI-CIQ-SHP version combines the WPAI with classroom impairment and specific health problems questions. The questionnaire consists of ten items scored on a scale of 0–10, with 0 indicating no effect and 10 indicating that health condition or concern completely prevented the individual from working or performing in school. The WPAI-CIQ-SHP yields scores on absenteeism (proportion of work hours missed), presenteeism (degree that health affected productivity while working), overall work or school impairment (linear combination of absenteeism and presenteeism), and activity impairment (degree to which health affected regular activities). The WPAI-CIQ-SHP scores are expressed as impairment percentages, with higher numbers indicating greater impairment and less productivity [10].

**Hospital Anxiety and Depression Scale (HADS):** The HADS^®^ comprises the following two subscales: 1 Anxiety scale (seven items); 2 Depression scale (seven items) [11]. Each item is scored from 0 to 3 resulting in a score range from 0 to 21 for each subscale, with higher scores indicating more severe anxiety or depression [11].

# Table S1. Asynchronous forum inclusion and exclusion criteria

| Inclusion | | Exclusion |
| --- | --- | --- |
| - Aged ≥18 years - Ability to communicate proficiently in English - Either: - Diagnosis of a medical condition reducing the immunity or making the participants clinically extremely vulnerable to severe COVID-19 (e.g., chronic kidney disease, chronic obstructive pulmonary disease, hepatocellular carcinoma, lupus, kidney transplant)   OR   - Be a caregiver of an individual with a diagnosis of a medical condition reducing the immunity or making the individual clinically extremely vulnerable to severe COVID-19 - Willing and able to participate in the study | - Significant disability, limited legal capacity, or any other lack of fitness, which, in the opinion of the study team, would significantly hinder the participant’s involvement or ability to complete the interview | |

*COVID-19* coronavirus disease 2019

# Table S2. Characteristics of participants in the asynchronous forums (*n* = 24)

| Characteristic, *n* (%) | Adults at high risk of severe COVID-19 outcomes  (*n* = 21) | Caregivers  (*n* = 3) |
| --- | --- | --- |
| Sex |  |  |
| Female | 13 (62) | 3 (100) |
| Participation in asynchronous forum sessions |  |  |
| Both forums | 19 (90) | 2 (67) |
| Initial online forum only | 1 (5) | 1 (33) |
| Follow-up forum only | 1 (5) | 0 |
| Country of origin |  |  |
| United States | 7 (33) | 2 (67) |
| United Kingdom | 8 (38) | 0 |
| Canada | 5 (24) | 0 |
| Singapore | 0 | 1 (33) |
| Australia | 1 (5) | 0 |
| High-risk condition |  |  |
| End-stage kidney disease | 7 (33) | 2 (67)^a^ |
| Cancer (generic) | 4 (19) | 1 (33)^a^ |
| Chronic obstructive pulmonary disease | 4 (19) | 0 |
| Systemic lupus erythematosus | 2 (10) | 0 |
| Non-alcoholic steatohepatitis | 2 (10) | 0 |
| Eosinophilic granulomatosis with polyangiitis | 1 (5) | 0 |
| Heart failure | 1 (5) | 0 |

^a^Indicates the condition of the person for whom the caregiver provides care.

# Table S3. EAGLE Study inclusion and exclusion criteria

|  | Adults (age ≥18 years) | Adolescents (age 13–17 years) Children (age 6 months–12 years) | |
| --- | --- | --- | --- |
| Inclusion criteria | | | |
| Individuals with immunocompromising conditions | - At least 1 specified immunocompromising condition or treatment within 2 months prior to study enrollment | | - At least 1 specified immunocompromising condition or treatment within 2 months prior to study enrollment |
| Individuals without immunocompromising conditions | - No specified immunocompromising conditions, treatments, or history of an immunocompromising condition or treatment since January 2020 | | - No specified immunocompromising conditions, treatments, or history of an immunocompromising condition or treatment since January 2020 |
| Caregivers of  children or adolescents | - Formal caregiver (i.e., a parent or legal guardian) of the child included in the study - Lives (at least some of the time) with the child included in the study - If caregiver’s child is ≥5 years: willing and able to provide consent for their child to participate in the study in addition to assent from the child or adolescent | | - N/A |
| All individuals | - Age ≥18 years - Resides in one of the eligible study countries - Able to read and understand English (US and UK) or Spanish (US) - Willing to complete a 30- to 45-minute online survey related to feelings, health, living situation, and other descriptive questions, within one week of the survey becoming available to them - Willing and able to provide consent to participate in the study | | - Age 6 months to <18 years - Resides in one of the eligible study countries - Must have a formal caregiver who meets caregiver eligibility criteria - For children ≥5 years:   - Able to understand English (US and UK) or Spanish (US) at a level typical for their age, with caregiver assistance if needed   - Willing and able to complete questions appropriate for their age about feelings and health, and simple descriptive questions lasting less than 15 minutes, with caregiver assistance if needed   - Willing and able to provide assent to participate in the study |
| Exclusion criteria | | | |
| Any individual was excluded from participating in the study if any of the following applied:   - Participating in clinical trial for experimental/investigational treatments for immunocompromising conditions and/or preventions or treatments of SARS-CoV-2 infection and/or COVID-19 - Hospitalized or admitted to an inpatient facility at recruitment - History of administration of tixagevimab/cilgavimab or any other passive immunization therapy for COVID-19 (history of vaccination against COVID-19 is acceptable) | | | |

*COVID-19* coronavirus disease 2019; *N/A* not applicable; *SARS-CoV-2* severe acute respiratory syndrome coronavirus 2

# **Table S4**. Clinical outcome assessments used in the EAGLE Study by age

|  | Non-adults (with assistance from caregivers, if needed) | | | | | | | | | | | | |
| --- | --- | --- | --- | --- | --- | --- | --- | --- | --- | --- | --- | --- | --- |
| COA* | Adults  (≥18 years) | | Adolescents (13–17 years) | | Older children (8–12 years) | | Young children (5–7 years) | | Toddlers  (2–4 years) | | Infants (0.5– <2 years) | Caregivers (≥18 years) | |
| PDS-C19 | ✓ | ✓ | | ✓^d^ | | ✓^d^ | | ✓^d^ | | ✓^d^ | | | ✓^e^ |
| DMOL | ✓ | ✓ | | ✓ | |  | |  | |  | | |  |
| WPAI-CIQ-SHP^a^ | ✓ | ✓^c^ | |  | |  | |  | |  | | | ✓^e^ |
| HADS | ✓ | ✓ | |  | |  | |  | |  | | | ✓^e^ |
| QDIS-7^a^ | ✓ |  | |  | |  | |  | |  | | |  |
| Generic HRQoL |  |  | |  | |  | |  | |  | | |  |
| SF-12v2 | ✓ |  | |  | |  | |  | |  | | |  |
| PedsQL Generic Core |  | ✓ | | ✓ | | ✓ | | ✓^d^ | |  | | |  |
| PedsQL Infant |  |  | |  | |  | |  | | ✓^d^ | | |  |
| PedsQL FIM |  |  | |  | |  | |  | |  | | | ✓^e^ |
| Health State Utilities |  |  | |  | |  | |  | |  | | |  |
| SF-6D^b^ | ✓ |  | |  | |  | |  | |  | | |  |
| EQ-5D-5L | ✓ | ✓ | |  | |  | |  | |  | | | ✓^e^ |
| EQ-5D-Y |  |  | | ✓ | | ✓^d^ | |  | |  | | |  |

^a^Items included the specific attribution to the following condition or health problem: “avoiding COVID-19.”

^b^SF-6D is derived from the SF-12v2 instrument.

^c^Only school-related questions administered.

^d^Solely proxy-reported, proxy version 1 for EQ-5D-Y (caregiver’s opinion).

^e^Reported for themselves, not as a proxy.

**DMOL* Direct Measure of Loneliness; *EQ-5D-5L* 5-level version of the EQ-5D; *EQ-5D-Y* Child-Friendly Version of the EQ-5D; *HADS* Hospital Anxiety and Depression Scale; *PDS-C19* Physical Distancing Scale for COVID-19 Avoidance; *PedsQL* Pediatric Quality of Life Inventory; PedsQLFIM, PedsQL Family Impact Module; *QDIS-7* Quality-of-Life Disease Impact Scale 7; *SF-12v2* 12-Item Short Form Health Survey Version 2; *SF-6D* Short Form 6-Dimensions; *WPAI-CIQ-SHP* Work Productivity and Activity Impairment Questionnaire plus Classroom Impairment Questions: Specific Health Problem

# Table S5. Frequency item response distribution for the final draft of the PDS-C19 (frequency of avoidant behaviors), validation sample (*n* = 1059)

|  |  | Frequency, *n* (%) | | | | |
| --- | --- | --- | --- | --- | --- | --- |
| Item | Label | Never | Rarely | Sometimes | Often | Always |
| 1 | Family (6 ft) | 474 (44.8) | 202 (19.1) | 216 (20.4) | 105 (9.9) | 62 (5.9) |
| 2 | Pass by (6 ft) | 288 (27.2) | 182 (17.2) | 245 (23.1) | 218 (20.6) | 126 (11.9) |
| 3 | Casual interaction (6 ft) | 332 (31.4) | 209 (19.7) | 266 (25.1) | 176 (16.6) | 76 (7.2) |
| 4 | Essential travel | 517 (48.8) | 181 (17.1) | 191 (18.0) | 125 (11.8) | 45 (4.2) |
| 5 | Non-essential travel | 416 (39.3) | 174 (16.4) | 225 (21.2) | 145 (13.7) | 99 (9.3) |
| 6 | Visitors | 422 (39.8) | 169 (16.0) | 212 (20.0) | 157 (14.8) | 99 (9.3) |
| 7 | Outdoors | 435 (41.1) | 173 (16.3) | 205 (19.4) | 153 (14.4) | 93 (8.8) |
| 8 | Small crowds | 444 (41.9) | 210 (19.8) | 231 (21.8) | 117 (11.0) | 57 (5.4) |
| 9 | Large crowds | 344 (32.5) | 154 (14.5) | 240 (22.7) | 188 (17.8) | 133 (12.6) |

*PDS-C19* Physical Distancing Scale for COVID-19 Avoidance

# **Table S6.** Duration item response distribution for the final draft of the PDS-C19 (onset of current avoidant behaviors), validation sample (*n* = 1059)

|  | | Onset of current behavior n (%) | | | | | | |
| --- | --- | --- | --- | --- | --- | --- | --- | --- |
| Item | Label | Not currently doing | <1 month | 1 to 6  months | 6 months to 1 year | 1 to 2 years | Since start  of pandemic | Precedes pandemic |
| 1 | Family (6 ft) | 586 (55.3) | 35 (3.3) | 48 (4.5) | 59 (5.6) | 58 (5.5) | 248 (23.4) | 25 (2.4) |
| 2 | Pass by (6 ft) | 398 (37.6) | 38 (3.6) | 57 (5.4) | 69 (6.5) | 89 (8.4) | 366 (34.6) | 42 (4.0) |
| 3 | Casual interaction (6 ft) | 478 (45.1) | 38 (3.6) | 55 (5.2) | 63 (5.9) | 83 (7.8) | 307 (29.0) | 35 (3.3) |
| 4 | Essential travel | 600 (56.7) | 27 (2.5) | 52 (4.9) | 62 (5.9) | 58 (5.5) | 235 (22.2) | 25 (2.4) |
| 5 | Non-essential travel | 507 (47.9) | 30 (2.8) | 57 (5.4) | 69 (6.5) | 65 (6.1) | 299 (28.2) | 32 (3.0) |
| 6 | Visitors | 526 (49.7) | 34 (3.2) | 46 (4.3) | 66 (6.2) | 67 (6.3) | 278 (26.3) | 42 (4.0) |
| 7 | Outdoors | 555 (52.4) | 34 (3.2) | 58 (5.5) | 64 (6.0) | 67 (6.3) | 256 (24.2) | 25 (2.4) |
| 8 | Small crowds | 567 (53.5) | 29 (2.7) | 45 (4.2) | 71 (6.7) | 69 (6.5) | 252 (23.8) | 26 (2.5) |
| 9 | Large crowds | 436 (41.2) | 32 (3.0) | 59 (5.6) | 48 (4.5) | 81 (7.6) | 357 (33.7) | 46 (4.3) |

*PDS-C19* Physical Distancing Scale for COVID-19 Avoidance

# **Table S7**. Polychoric correlations between the nine final-draft PDS-C19 frequency items, validation sample (*n* = 1059)

|  |  | Item | | | | | | | | |
| --- | --- | --- | --- | --- | --- | --- | --- | --- | --- | --- |
| Item | Label | 1 | 2 | 3 | 4 | 5 | 6 | 7 | 8 | 9 |
| 1 | Family (6 ft) | – |  |  |  |  |  |  |  |  |
| 2 | Pass by (6 ft) | 0.73 | – |  |  |  |  |  |  |  |
| 3 | Casual interaction (6 ft) | 0.79 | 0.88 | – |  |  |  |  |  |  |
| 4 | Essential travel | 0.75 | 0.73 | 0.75 | – |  |  |  |  |  |
| 5 | Non-essential travel | 0.74 | 0.79 | 0.82 | 0.83 | – |  |  |  |  |
| 6 | Visitors | 0.76 | 0.81 | 0.84 | 0.80 | 0.87 | – |  |  |  |
| 7 | Outdoors | 0.74 | 0.78 | 0.80 | 0.79 | 0.82 | 0.86 | – |  |  |
| 8 | Small crowds | 0.76 | 0.79 | 0.80 | 0.83 | 0.84 | 0.85 | 0.86 | – |  |
| 9 | Large crowds | 0.71 | 0.82 | 0.82 | 0.77 | 0.86 | 0.86 | 0.83 | 0.86 | – |

*PDS-C19* Physical Distancing Scale for COVID-19 Avoidance

# **Table S8**. Polychoric correlations between the nine final-draft PDS-C19 frequency items (excluding those who answered “Never” to all items, *n* = 837)^a^

|  |  | Item | | | | | | | | |
| --- | --- | --- | --- | --- | --- | --- | --- | --- | --- | --- |
| Item | Label | 1 | 2 | 3 | 4 | 5 | 6 | 7 | 8 | 9 |
| 1 | Family (6 ft) | – |  |  |  |  |  |  |  |  |
| 2 | Pass by (6 ft) | 0.54 | – |  |  |  |  |  |  |  |
| 3 | Casual interaction (6 ft) | 0.66 | 0.78 | – |  |  |  |  |  |  |
| 4 | Essential travel | 0.63 | 0.56 | 0.60 | – |  |  |  |  |  |
| 5 | Non-essential travel | 0.59 | 0.62 | 0.69 | 0.75 | – |  |  |  |  |
| 6 | Visitors | 0.64 | 0.66 | 0.73 | 0.70 | 0.79 | – |  |  |  |
| 7 | Outdoors | 0.61 | 0.62 | 0.66 | 0.68 | 0.71 | 0.78 | – |  |  |
| 8 | Small crowds | 0.64 | 0.63 | 0.67 | 0.75 | 0.75 | 0.77 | 0.79 | – |  |
| 9 | Large crowds | 0.54 | 0.66 | 0.68 | 0.63 | 0.76 | 0.76 | 0.71 | 0.77 | – |

^a^9-item centering sample.

*PDS-C19* Physical Distancing Scale for COVID-19 Avoidance

# **Table S9**. EFA – fit statistics of the nine-item final draft of the PDS-C19, EFA sample (*n* = 542)

| Factor model | Number of parameters | χ^2^ | df | *p*-value | Absolute Fit | |  | | Comparative Fit | |
| --- | --- | --- | --- | --- | --- | --- | --- | --- | --- | --- |
|  |  |  |  |  | RMSEA (90% CI) | SRMR |  | | CFI | TLI |
| Single | 9 | 274.08 | 27 | <0.0001 | 0.13 (0.12–0.14) | 0.03 |  | 0.99 | | 0.99 |
| Bifactor  (1 general,  2 specific) | 24 | 58.67 | 12 | <0.0001 | 0.09 (0.06–0.11) | 0.01 |  | 1.00 | | 1.00 |

*CFI* comparative fix index; *CI* confidence interval; *df* degrees of freedom; *EFA* exploratory factor analysis; *PDS-C19* Physical Distancing Scale for COVID-19 Avoidance; *RMSEA* root mean square error of approximation; *SRMR* standardized root mean residual; *TLI* Tucker-Lewis index; *χ^2^* Chi-square test

# Table S10. MIMIC model DIF analysis for Physical Distancing Scale (PDS-C19) intensity items: Continuous Age - Psychometric validation set

| **Item** | **Non-uniform (interaction)** | | | | |  | **Uniform (main effect)** | | | | |
| --- | --- | --- | --- | --- | --- | --- | --- | --- | --- | --- | --- |
|  | **Estimate** | **SE** | ***p*** | ***p_adj_*** | ***β*** |  | **Estimate** | **SE** | ***p*** | ***p_adj_*** | ***β*** |
| 1. Avoid within 6 ft people I care about | –0.09 | 0.05 | 0.092 | 0.184 | –0.08 |  | 0.03 | 0.04 | 0.385 | 0.385 | 0.03 |
| 3. Avoid within 6 ft casual interaction | –0.23 | 0.05 | 0.000 | **0.000** | –0.16 |  | 0.12 | 0.04 | 0.003 | **0.024** | 0.09 |
| 4. Avoid essential travel | –0.00 | 0.06 | 0.974 | 0.974 | –0.00 |  | –0.06 | 0.04 | 0.178 | 0.237 | –0.04 |
| 5. Avoid non-essential travel | 0.03 | 0.06 | 0.660 | 0.880 | 0.02 |  | –0.05 | 0.05 | 0.331 | 0.378 | –0.03 |
| 6. Avoid visitors | 0.14 | 0.06 | 0.021 | 0.084 | 0.08 |  | –0.08 | 0.04 | 0.075 | 0.150 | –0.04 |
| 7. Avoid outdoor gatherings | 0.15 | 0.07 | 0.032 | 0.085 | 0.09 |  | –0.09 | 0.06 | 0.095 | 0.152 | –0.06 |
| 8. Avoid places with few people | 0.12 | 0.07 | 0.106 | 0.170 | 0.07 |  | –0.09 | 0.05 | 0.074 | 0.197 | –0.05 |
| 9. Avoid crowded places | 0.01 | 0.06 | 0.829 | 0.947 | 0.01 |  | 0.11 | 0.04 | 0.005 | **0.020** | 0.07 |
| *Β* standardized regression coefficient*; DIF* differential item functioning; *MIMIC* multiple indicators multiple cause; *P_adj_*, adjusted *P* value (stepped-up for analyzing eight items); *PDS-C19* Physical Distancing Scale for COVID-19 Avoidance; *SE* standard error | | | | | | | | | | | |

# **Table S11**. MIMIC model for differential item functioning analysis for final-draft PDS-C19 intensity item 3 (Casual interaction [6 ft]) and item 9 (Crowded place), by age group (Reference: Adult), CFA sample (*n* = 517)

| Subgroup | Non-uniform (interaction) | | | | |  | Uniform (main effect) | | | | |
| --- | --- | --- | --- | --- | --- | --- | --- | --- | --- | --- | --- |
|  | Estimate | SE | *p*-value | *p_adj_* | *β* |  | Estimate | SE | *p*-value | *p_adj_* | *β* |
| **3: Avoid within 6 ft casual interaction** | | | | | | | | | | | |
| Caregiver (≥18 years)^a^ | 1.35 | 0.51 | 0.008 | **0.024** | 0.33 |  | −0.81 | 0.30 | 0.008 | **0.048** | −0.19 |
| Adolescent (13–17 years) | 0.97 | 0.42 | 0.020 | **0.024** | 0.24 |  | −0.86 | 0.33 | 0.009 | **0.027** | −0.21 |
| Older child (8–12 years)^b^ | 1.54 | 0.45 | 0.001 | **0.006** | 0.38 |  | −0.50 | 0.33 | 0.128 | 0.192 | −0.12 |
| Younger child (5–7 years) ^b^ | 2.40 | 0.94 | 0.011 | **0.022** | 0.59 |  | −1.29 | 0.61 | 0.035 | 0.070 | −0.31 |
| Toddler (2–4 years) ^b^ | 0.85 | 0.69 | 0.218 | 0.218 | 0.21 |  | −0.61 | 0.55 | 0.262 | 0.262 | −0.15 |
| Infant (0.5–2 years) ^b^ | 2.56 | 1.05 | 0.014 | **0.021** | 0.63 |  | −0.90 | 0.71 | 0.206 | 0.247 | −0.22 |
| **9: Avoid crowded places** | | | | | | | | | | | |
| Caregiver (≥18 years) ^a^ | –0.42 | 0.60 | 0.488 | 0.732 | –0.09 |  | –1.19 | 0.37 | 0.001 | **0.006** | –0.24 |
| Adolescent (13-17 years) | –0.49 | 0.49 | 0.317 | 0.634 | –0.10 |  | 0.20 | 0.36 | 0.571 | 0.685 | 0.04 |
| Older child (8–12 years)^b^ | –0.07 | 0.62 | 0.909 | 0.909 | –0.01 |  | –0.52 | 0.39 | 0.184 | 0.276 | –0.10 |
| Younger child (5–7 years)^b^ | –1.02 | 0.48 | 0.032 | 0.192 | –0.21 |  | –0.16 | 0.40 | 0.689 | 0.689 | –0.03 |
| Toddler (2–4 years) ^b^ | 1.70 | 1.43 | 0.234 | 0.702 | 0.35 |  | –1.34 | 0.99 | 0.175 | 0.350 | –0.27 |
| Infant (0.5–2 years) ^b^ | 0.57 | 1.12 | 0.608 | 0.730 | 0.12 |  | –1.15 | 0.46 | 0.011 | **0.033** | –0.23 |

^a^Reported for themselves, not as a proxy.

^b^Solely proxy-reported (caregiver’s opinion).

For each item DIF was assessed via a MIMIC model: After regressing the covariate (DIF variable) on the latent PDS-C19 score, the interaction between the covariate and the latent PDS-C19 score was regressed on a given item (moderation) to assess non-uniform DIF and the covariate as a main effect was regressed on the given item to test for uniform DIF.

Estimate denotes the magnitude of the effect on the item’s 1–5 scale; *β* denotes the magnitude in SD units of the latent PDS scale (*β* = 0.20, 0.50, and 0.80 for small, medium, and large effects, respectively).

For a given item, each focal group was tested separately, with *p* denoting the unadjusted *p*-value, and *p_adj_*, which is the *p*-value multiplied by the count of focal groups divided by the *p*-value’s descending ordinal rank, denoting a stepped-up (Benjamin-Hochberg) value to correct for multiple-testing.

*Β* standardized regression coefficient*; CFA* confirmatory factor analysis*; DIF* differential item functioning; *MIMIC* multiple-indicators, multiple cause model; *p_adj_* adjusted *p*-value; *PDS-C19* Physical Distancing Scale for COVID-19 Avoidance; SD standard deviation; *SE* standard error

# **Table S12**. PDS-C19 CFA of the final draft of the PDS-C19 – fit statistics for selected items, CFA sample (*n* = 517)

|  |  | |  |  |  | Absolute Fit | | | Comparative Fit | | |
| --- | --- | --- | --- | --- | --- | --- | --- | --- | --- | --- | --- |
| Factor Model | Parameters | χ^2^ | | df | *p-*value | RMSEA  (90% CI) | SRMR |  |  | CFI | TLI |
| Full PDS-C19 | 45 | 274.83 | | 27 | <0.0001 | 0.13 (0.12−0.15) | 0.02 |  |  | 0.99 | 0.99 |
| Drop Item 2 | 40 | 144.42 | | 20 | <0.0001 | 0.11 (0.09−0.13) | 0.02 |  |  | 0.99 | 0.99 |
| Drop Item 3 | 40 | 117.11 | | 20 | <0.0001 | 0.10 (0.08−0.11) | 0.01 |  |  | 1.00 | 0.99 |
| Drop Items 2 and 3 | 35 | 91.39 | | 14 | <0.0001 | 0.10 (0.08−0.12) | 0.01 |  |  | 1.00 | 0.99 |

*CFA,* confirmatory factor analysis; *CFI* comparative fix index; *CI* confidence interval; *df* degrees of freedom; *PDS-C19* Physical Distancing Scale for COVID-19 Avoidance; *RMSEA* root mean square error of approximation; *SRMR* standardized root mean residual; *TLI* Tucker Lewis index; *χ*^2^ Chi square test

# Table S13. CFA: WLSMV standardized item loadings and thresholds of the final draft of the PDS-C19, confirmatory factor analysis sample (*n* = 517)

| Item | Label | Loading (λ) | Thresholds | | | | | | | |
| --- | --- | --- | --- | --- | --- | --- | --- | --- | --- | --- |
|  |  |  | 1 | 2 | | 3 | | 4 | |  |
| 1 | Family (6 ft) | 0.79 | −0.10 | | 0.33 | | 1.07 | | 1.64 | |
| 4 | Essential travel | 0.87 | −0.04 | | 0.40 | | 0.99 | | 1.70 | |
| 5 | Non-essential travel | 0.92 | −0.25 | | 0.17 | | 0.80 | | 1.38 | |
| 6 | Visitors | 0.93 | −0.25 | | 0.14 | | 0.76 | | 1.30 | |
| 7 | Outdoors | 0.91 | −0.21 | | 0.21 | | 0.78 | | 1.29 | |
| 8 | Small crowds | 0.93 | −0.23 | | 0.30 | | 0.91 | | 1.66 | |
| 9 | Large crowds | 0.92 | −0.44 | | −0.05 | | 0.49 | | 1.15 | |

Note. Dividing a given threshold by its loading yields the location where giving that response value or lower has the same predicted probability as that of giving a higher response value (this corresponds to the difficulty parameter in a graded response model)

*CFA,* confirmatory factor analysis; *PDS-C19* Physical Distancing Scale for COVID-19 Avoidance; *WLSMV* weighted least squares mean and variance

# Table S14. PDS-C19 internal consistency reliability: Overlap-corrected item-total polyserial correlations (centering sample, i.e., excluding those who answered “Never” to all items, *n* = 796)

| Item | Label | Correlation |
| --- | --- | --- |
| 1 Family (6 ft) | Avoid people cared about | 0.61 |
| 4 Essential travel | Avoid essential travel | 0.74 |
| 5 Non-essential travel | Avoid non-essential travel | 0.79 |
| 6 Visitors | Avoid having visitors | 0.82 |
| 7 Outdoors | Avoid outdoor gatherings | 0.78 |
| 8 Small crowds | Avoid places where expect few people | 0.81 |
| 9 Large crowds | Avoid places where expect many people | 0.74 |

*PDS-C19* Physical Distancing Scale for COVID-19 Avoidance

# Table S15. Summary of physical distancing scales used in other studies

| Author | Details of Scale |
| --- | --- |
| Prachthauser *et al.*[12] | 14-item scale to measure adherence to prevention strategies during pandemic in undergraduate students |
| Islam *et al.*[13] | Binary-coded scale used to compare adherence to COVID-19 preventive measures among immunocompromised adults with non-immunocompromised adults during the COVID-19 pandemic |
| Gouin *et al.*[14] | Study used socio-demographic, health, cognitive, emotional, and social factors during the COVID-19 pandemic to predict binary physical distancing adherence derived from a five-item scale among adults living in Quebec |
| Chung *et al.*[15] | Scale originally developed by Gouin *et al* (with two additional items appended related to mask-wearing) was used to examine the mediation effect of viral anxiety on healthcare workers’ adherence to physical distancing during the COVID-19 pandemic |
| Hong *et al*.[16] | Scale originally developed by Gouin *et al* examined the mediating effect of injunctive norms on the degree to which health beliefs and viral anxiety predict adherence to COVID-19-related physical distancing in the general population |
| Hammoudi *et al*.[17] | Scale originally developed by Gouin *et al* examined the mediating effect of personal injunctive norms on the degree to which health beliefs predicted adherence to COVID-19–related physical distancing in individuals with cancer |

*COVID-19* coronavirus disease 2019

Fig. S1. Overview of development and validation stages of the PDS-C19

*PDS-C19* Physical Distancing Scale for COVID-19 Avoidance

# **Fig. S2**. T-score distribution of seven final PDS-C19 frequency items (centering sample, i.e., excluding those who answered “Never” to all items, *n* = 796)

*PDS-C19* Physical Distancing Scale for COVID-19 Avoidance; *SD* standard deviation

# References

1.	Ware, J., Jr., Kosinski, M., & Keller, S. D. (1996). A 12-Item Short-Form Health Survey: construction of scales and preliminary tests of reliability and validity. *Medical Care, 34*(3), 220-233. 10.1097/00005650-199603000-00003

2.	Ware, J. E., Jr,. Quality of Life Disease Impact Scale–7 item scale (QDIS-7-item scale). Retrieved March 9, 2024, from <https://eprovide.mapi-trust.org/instruments/quality-of-life-disease-impact-scale-7-item-scale>

3.	Office for National Statistics. Recommended national indicators of loneliness. Retrieved March 9, 2024, from <https://www.ons.gov.uk/peoplepopulationandcommunity/wellbeing/compendium/nationalmeasurementofloneliness/2018/recommendednationalindicatorsofloneliness>

4.	Varni, J. W., Seid, M., & Kurtin, P. S. (2001). PedsQL^TM^ 4.0: reliability and validity of the Pediatric Quality of Life Inventory^TM^ version 4.0 generic core scales in healthy and patient populations. *Medical Care, 39*(8), 800-812. <https://doi.org/10.1097/00005650-200108000-00006>

5.	Varni, J. W., Limbers, C. A., Neighbors, K., Schulz, K., Lieu, J. E., Heffer, R. W., Tuzinkiewicz, K., Mangione-Smith, R., Zimmerman, J. J., & Alonso, E. M. (2011). The PedsQL^TM^ Infant Scales: feasibility, internal consistency reliability, and validity in healthy and ill infants. *Quality of Life Research, 20*(1), 45-55. <https://doi.org/10.1007/s11136-010-9730-5>

6.	Varni, J. W., Sherman, S. A., Burwinkle, T. M., Dickinson, P. E., & Dixon, P. (2004). The PedsQL^TM^ Family Impact Module: preliminary reliability and validity. *Health and Quality of Life Outcomes, 2*, 55. <https://doi.org/10.1186/1477-7525-2-55>

7.	Herdman, M., Gudex, C., Lloyd, A., Janssen, M., Kind, P., Parkin, D., Bonsel, G., & Badia, X. (2011). Development and preliminary testing of the new five-level version of EQ-5D (EQ-5D-5L). *Quality of Life Research, 20*(10), 1727-1736. <https://doi.org/10.1007/s11136-011-9903-x>

8.	EuroQoL Group. EQ-5D. Retrieved March 9, 2024, from <https://euroqol.org/eq-5d-instruments/eq-5d-y-about/>

9.	Brazier, J., Roberts, J., & Deverill, M. (2002). The estimation of a preference-based measure of health from the SF-36. *Journal of Health Economics, 21*(2), 271-292. <https://doi.org/10.1016/s0167-6296(01)00130-8>

10.	Reilly, M. C., Zbrozek, A. S., & Dukes, E. M. (1993). The validity and reproducibility of a work productivity and activity impairment instrument. *Pharmacoeconomics, 4*(5), 353-365. <https://doi.org/10.2165/00019053-199304050-00006>

11.	Zigmond, A. S., & Snaith, R. P. (1983). The Hospital Anxiety and Depression Scale. *Acta Psychiatrica Scandinavica, 67*(6), 361-370. <https://doi.org/10.1111/j.1600-0447.1983.tb09716.x>

12.	Prachthauser, M., Cassisi, J. E., Le, T. A., & Nicasio, A. V. (2020). The Social Distance Scale (v1): a screening instrument to assess patient adherence to prevention strategies during pandemics. *International Journal of Environmental Research and Public Health, 17*(21), <https://doi.org/10.3390/ijerph17218158>

13.	Islam, J. Y., Vidot, D. C., Havanur, A., & Camacho-Rivera, M. (2021). Preventive behaviors and mental health-related symptoms among immunocompromised adults during the COVID-19 pandemic: an analysis of the COVID impact survey. *AIDS Research and Human Retroviruses, 37*(4), 304-313. <https://doi.org/10.1089/AID.2020.0302>

14.	Gouin, J. P., MacNeil, S., Switzer, A., Carrese-Chacra, E., Durif, F., & Knauper, B. (2021). Socio-demographic, social, cognitive, and emotional correlates of adherence to physical distancing during the COVID-19 pandemic: a cross-sectional study. *Canadian Journal of Public Health, 112*(1), 17-28. <https://doi.org/10.17269/s41997-020-00457-5>

15.	Chung, S., Lee, T., Hong, Y., Ahmed, O., Silva, W. A. D., & Gouin, J. P. (2022). Viral anxiety mediates the influence of intolerance of uncertainty on adherence to physical distancing among healthcare workers in COVID-19 pandemic. *Frontiers in Psychiatry, 13*, 839656. <https://doi.org/10.3389/fpsyt.2022.839656>

16.	Hong, Y., An, H., Cho, E., Ahmed, O., Ahn, M. H., Yoo, S., & Chung, S. (2023). Psychometric properties of the Korean version of questionnaires on adherence to physical distancing and health beliefs about COVID-19 in the general population. *Frontiers in Psychiatry, 14*, 1132169. <https://doi.org/10.3389/fpsyt.2023.1132169>

17.	Hammoudi, S. F., Ahmed, O., An, H., Hong, Y., Ahn, M. H., & Chung, S. (2023). Adherence to physical distancing and health beliefs about COVID-19 among patients with cancer. *Journal of Korean Medical Science, 38*(43), e336. <https://doi.org/10.3346/jkms.2023.38.e336>
